# Synthesis of Polyampholyte Janus‐like Microgels by Coacervation of Reactive Precursors in Precipitation Polymerization

**DOI:** 10.1002/anie.201910450

**Published:** 2019-12-10

**Authors:** Wenjing Xu, Andrey Rudov, Alex Oppermann, Sarah Wypysek, Michael Kather, Ricarda Schroeder, Walter Richtering, Igor I. Potemkin, Dominik Wöll, Andrij Pich

**Affiliations:** ^1^ DWI—Leibniz-Institute for Interactive Materials e.V. RWTH-Aachen University Forckenbeckstraße 50 52074 Aachen Germany; ^2^ Institute of Technical and Macromolecular Chemistry RWTH Aachen University Worringerweg 2 52074 Aachen Germany; ^3^ Institute of Physical Chemistry RWTH Aachen University Landoltweg 2 52074 Aachen Germany; ^4^ Physics Department Lomonosov Moscow State University Moscow 119991 Russian Federation; ^5^ National Research South Ural State University Chelyabinsk 454080 Russian Federation; ^6^ Aachen Maastricht Institute for Biobased Materials (AMIBM) Maastricht University Brightlands Chemelot The Netherlands

**Keywords:** Janus-like microgels, molecular modeling, polyampholyte, responsive materials, self-assembly

## Abstract

Controlling the distribution of ionizable groups of opposite charge in microgels is an extremely challenging task, which could open new pathways to design a new generation of stimuli‐responsive colloids. Herein, we report a straightforward approach for the synthesis of polyampholyte Janus‐like microgels, where ionizable groups of opposite charge are located on different sides of the colloidal network. This synthesis approach is based on the controlled self‐assembly of growing polyelectrolyte microgel precursors during the precipitation polymerization process. We confirmed the morphology of polyampholyte Janus‐like microgels and demonstrate that they are capable of responding quickly to changes in both pH and temperature in aqueous solutions.

## Graphical abstract

**Polyampholyte Janus‐like microgels** are synthesized using the controlled, electrostatically driven self‐assembly and coacervation of growing, oppositely charged microgel precursors during precipitation polymerization. This enables the design of new drug carriers, switchable emulsion stabilizers, and adaptive catalyst carriers.

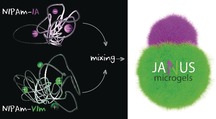

## Introduction

Microgels, which are defined as unique crosslinked macromolecular structures, fill the gap between macromolecules and colloids. They exhibit interesting properties like stimuli‐responsiveness, surface‐activity, and adaptability.^1^

The presence of charges in microgels makes them appealing for the development of drug‐delivery carriers,1a, 2 emulsion stabilizers,^3^ and functional coatings.^4^

A special class of charged microgels are polyampholyte microgels that carry opposite charges at different pH.^5^ Recently, polyampholyte microgels with a random and core–shell distribution of charges were synthesized by miniemulsion and precipitation polymerization.^6^ It has been shown that apart from their balance, the distribution of charges in microgels strongly influences the uptake and release of charged molecules.^7^

So far, no suitable method has been reported to synthesize polyampholyte Janus‐like microgels with oppositely charged faces. The attempts to synthesize Janus microgels were focused mainly on post‐modification methods, including masking techniques (surface adsorption/interface adsorption)^8^ or phase separation in microfluidic droplets.^9^

Dendukuri et al. reported the synthesis of Janus microgels by using a microfluidic device. Two non‐miscible fluids (monomers) were mixed with a photoinitiator to generate biphasic droplets, followed by UV‐triggered crosslinking.^10^ This method, however, could be applied only to a limited amount of monomers and cannot be used for the production of large quantities of Janus microgels.

Another approach to synthesize Janus colloids is the use of liquid–liquid interfaces for fixation and site‐specific post‐modification. H. Kawaguchi et al., for instance, prepared Janus microgels based on poly(*N*‐isopropylacrylamide) (PNIPAm) and acrylic acid (AAc).^11^ Microgels were adsorbed at the toluene–water interface, and amino groups were introduced via a carbodiimide coupling reaction on the side of the particles which are in contact with water. However, since microgels show a different swelling extension to different solvents, it is difficult to predict the portion of the surface being functionalized.

Furthermore, most methods for the synthesis of Janus microgels only functionalize the particle surface, while the microgel interior is unaffected. Concerning possible applications as drug carriers, the presence of functional groups in the microgel interior is desired, as it offers several advantages, such as improved solubility of the drug,^12^ protection against immunogenicity,^13^ and degradability.^14^ Furthermore, the directed self‐assembly of Janus colloids and multitude of their potential applications has raised high interest in the development of future advanced materials.8a With regard to this, the investigation on Janus microgels will potentially promote the understanding of their properties and the development of new programmed materials.8b, 15 Janus microgels show multiple functionalities and tunable interactions deriving from the polymeric motifs. They also allow responsive and adaptive interactions in bulk systems towards external stimuli and guest molecules that are needed for self‐assembly.^16^ In all, Janus microgels fulfill the concept of a well‐defined, versatile, multifunctional, and responsive material, and are therefore of profound interest both for fundamental research as well as for application toward advanced materials.

Existing approaches to Janus colloids16b, 17 are limited to certain chemical functionalities and cannot be transferred directly to microgels. Also, such synthesis methods cannot be performed on a large scale, prohibiting the production of Janus microgels in large amounts. The synthesis of Janus microgels by techniques that consider structure formation in a selective solvent or solvent mixtures like self‐assembly,^15^, ^18^ or phase separation,^19^ known for block‐copolymer assemblies, have not been reported so far.

**Scheme 1:**
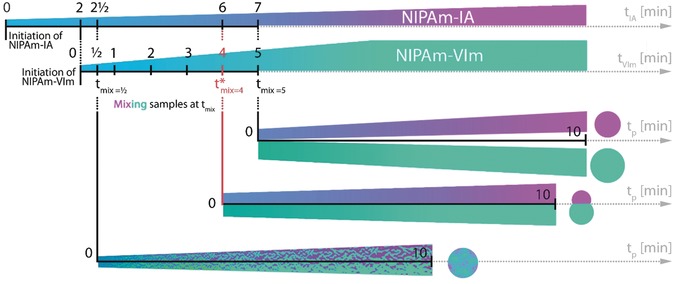
Synthesis route for microgels with various architectures and distributions of ionizable groups. When *t*_mix_<t*_mix_: microgels with a random distribution of ionizable groups are obtained, when *t*_mix_=t*_mix_: Janus‐like microgels are obtained, and when *t*_mix_>t*_mix_: separate microgels of opposite charges are obtained. t_IA_ is the polymerization time of NIPAm‐IA microgels, *t*_VIm_ is the polymerization time of NIPAm‐VIm microgels, *t*_mix_ is the time at which NIPAm‐IA and NIPAm‐VIm microgels were mixed, t*_mix_ is the time at which Janus‐like microgels were observed, and *t*_p_ is the polymerization time after mixing of the samples.

Herein, we present an easy and straightforward template‐free method for the synthesis of polyampholyte Janus‐like microgels with oppositely charged sides. This synthesis approach is based on the controlled electrostatically driven self‐assembly and coacervation of growing oppositely charged microgel precursors during precipitation polymerization. The key feature of the developed synthesis method is the fast mixing of two reaction mixtures with separately growing polyelectrolyte microgels carrying opposite charges at a specific reaction time (t*_mix_) and monomer conversion. We demonstrate that mixing two reaction mixtures at *t*_mix_<t*_mix_ leads to the formation of microgels with a random distribution of charges. Contrary, mixing two reaction mixtures at *t*_mix_>t*_mix_ leads to the formation of a mixture of separate polyanionic and polycationic microgels (Scheme 1). Controlling the distribution of ionizable groups within polyampholyte Janus‐like microgels enables the design of new efficient drug carriers, switchable emulsion stabilizers, and adaptive catalyst carriers. The developed synthesis method opens new possibilities to synthesize microgels with complex internal architecture using an industrially relevant and up‐scalable polymerization technique.

## Results and Discussion

To evolve a new synthesis approach for polyampholyte Janus‐like microgels, we decided to use *N*‐isopropylacrylamide (NIPAm) as the main monomer along with itaconic acid (IA) and 1‐vinylimidazole (VIm) as comonomers carrying ionizable groups of opposite charge. *N*,*N*′‐methylenebis(acrylamide) (BIS) was used as the crosslinker and 2,2′‐azobis[2‐methylpropionamidine] dihydrochloride (AMPA) as the initiator. NIPAm is probably the most widely used monomer for the synthesis of temperature‐responsive microgels.^20^ IA and VIm were used in our previous work for the synthesis of polyampholyte microgels with random6a and core–shell^20^ distribution of charges (amounts of ingredients are listed in the Supporting Information, Tables S2 and S3), and therefore were applied in the present study to enable their comparison with the properties of polyampholyte Janus‐like microgels.

The simplified design of the microgel synthesis developed in the present study is shown in Scheme 1. The precipitation polymerization of NIPAm‐IA and NIPAm‐VIm in water was initiated in two separated reaction vessels by the addition of the water‐soluble radical initiator (AMPA) at a reaction temperature of 70 °C (Supporting Information, Table S1). At certain reaction times, hot solutions with growing microgel precursors were rapidly mixed in a pre‐heated reaction vessel, and the polymerization was continued at 70 °C. As shown in Scheme 1, depending on the mixing time, polyampholyte microgels with a different distribution of ionizable groups and architectures could be obtained. The following parameters were varied to investigate their influence on the reaction process: 1) mixing time (*t*_mix_), 2) polymerization time after mixing (*t*_p_), 3) reaction temperature (before and after mixing), and 4) pH of reaction solutions. Accordingly, we found that a reaction temperature of 70 °C was most suitable in terms of polymerization rates and microgel yield. A pH of 6 was found to be optimal to maintain the colloidal stability of formed microgels, thus avoiding coagulate formation in the reactor.

In classical precipitation polymerization of NIPAm, the growing oligoradicals precipitate when they reach a critical chain length, forming microgel nuclei, which act as precursors for microgels.^21^ It is believed that starting from this point, the formed microgel precursors grow in size by the precipitation/crosslinking of newly formed polymer chains and reach the final size when all monomer is consumed. Based on these considerations, we decided to explore the early stage of the microgel precursor growth to find a suitable route to microgels with complex architectures.

The essential step was monitoring the precipitation copolymerization rate and microgel growth process for selected monomers. We used reaction calorimetry to monitor the polymerization heat and calculate conversion–time dependencies for microgel synthesis. Turbidity measurements and in situ dynamic light scattering (DLS) were applied to follow the nucleation and growth of microgels throughout the precipitation polymerization.

In the preliminary experiments, we performed three polymerization runs synthesizing NIPAm, NIPAm‐IA, and NIPAm‐VIm microgels under the same reaction conditions (monomer and initiator concentrations and reaction temperature). The addition of the initiator to the pre‐heated monomer solution set the start of the reaction. The calorimetric measurements of NIPAm and NIPAm‐VIm (Supporting Information, Figure S1) indicate that the precipitation polymerization for all polymerization runs is completed after 40 min, reaching high monomer conversions (95–100 %). Remarkably, the presence of comonomer VIm slows down the polymerization rate without strongly affecting the final monomer conversion, which was calculated based on the total polymerization heat. Unexpectedly, it was not possible to copolymerize NIPAm with IA in the calorimeter (though the reaction worked well in a double‐walled glass reactor), possibly due to a side reaction between IA and the metal parts of the reaction calorimeter. However, Erbil et al. demonstrated that by using free radical polymerization in 1,4‐dioxane, NIPAm units are considerably more reactive than IA units.^22^

**Figure 1:**
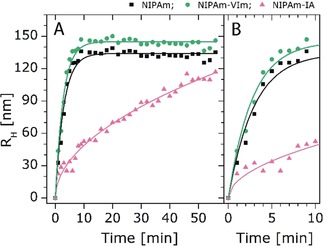
A) Time‐dependent DLS measurements for pure NIPAm (black square), NIPAm‐IA (pink triangle), and NIPAm‐VIm (green circle) microgels (measurements performed at 70 °C). B) Zoom‐in of (A) from 0 to 10 min. For all measurements, the particle growth during the polymerization process was fitted using the Boltzmann equation.

In situ time‐dependent DLS measurements for three polymerization runs visualize the microgel nucleation and growth stages (Figure 1). Pure NIPAm microgels reach a hydrodynamic radius R_H_ of approximately 140 nm after 5 min. NIPAm‐VIm microgels need a slightly longer reaction time of approximately 8 min to reach an R_H_ of approximately 150 nm. NIPAm‐IA microgels require the longest time (approximately 50 min) to reach their maximal size of approximately 110 nm. The in situ DLS data of NIPAm‐VIm correlate well with the calorimetric results presented in Figure S1 in the Supporting Information, showing that the presence of the comonomer VIm slows down microgel growth. An analogous statement can also be made for NIPAm‐IA, due to the much longer polymerization time required to reach a stable microgel size. This can be attributed to the increased hydrophilicity of oligoradicals containing VIm or IA monomer units and thus retarded precipitation onto the growing microgel nuclei. Another important conclusion from calorimetry and in situ DLS measurements is that the copolymerization processes of NIPAm with VIm and IA as well as the growth of microgels require different reaction times. Based on these experimental data, we hypothesize that rapid mixing of growing microgel nuclei carrying opposite charge at a specific reaction time may lead to the formation of polyampholyte Janus‐like microgels. Consequently, we narrowed down the most suitable mixing time between 0.5–5 min after the initiation of the polymerization, since both calorimetry and in situ DLS indicate that within this time frame, the microgel growth is fast.

Following this concept, we performed a series of polymerizations, as shown in Scheme 1, where the polymerization mixtures from two separate reaction vessels were mixed at different times (0.5–5 min). In these experiments, only the mixing time (*t*_mix_) and the polymerization time (t_p_) after mixing were varied.

For the first set of experiments, the synthesis of NIPAm‐VIm microgels was initiated 2 min after the initiation of NIPAm‐IA microgel synthesis. At *t*_mix_ of 0.5, 1, 2, 3, 4, and 5 min after initiation of NIPAm‐VIm, 1 mL of each dispersion was taken from separate reactors, and both samples were mixed immediately under continuous stirring at 70 °C. The respective mixtures were then allowed to continue to react for *t*_p_=1 h at 70 °C. Depending on *t*_mix_, colorless (small *t*_mix_) or milky (large *t*_mix_) dispersions were obtained. All synthesized microgels were purified by dialysis and subjected to careful characterization with transmission electron microscopy (TEM). To verify the formation of Janus‐like particles, the samples were stained with uranyl acetate U(Ac)_3_ first, given that U(Ac)_3_ binds selectively to the carboxyl groups of IA.

TEM images for a batch of samples prepared at *t*_p_=1 h are presented in Figure S2 in the Supporting Information. The images show that different *t*_mix_ lead to the formation of microgels with different distributions of IA. Mixing after 3 min results in some microgels with one side darker than the other, while the majority show microgels with a homogeneous distribution (Supporting Information, Figure S2 B,C). This shows that IA and VIm groups are spatially separated in some cases, proving the formation of Janus‐like microgels. However, as only a few Janus‐like microgels could be detected during the TEM measurements, the synthesis procedure needed to be optimized. One reasonable explanation for the observed paradox could be that if the polymerization time is too long, NIPAm monomer, which is in excess, builds up a shell around the “Janus‐core” thus preventing the observation of Janus‐like microgels through TEM.

**Figure 2:**
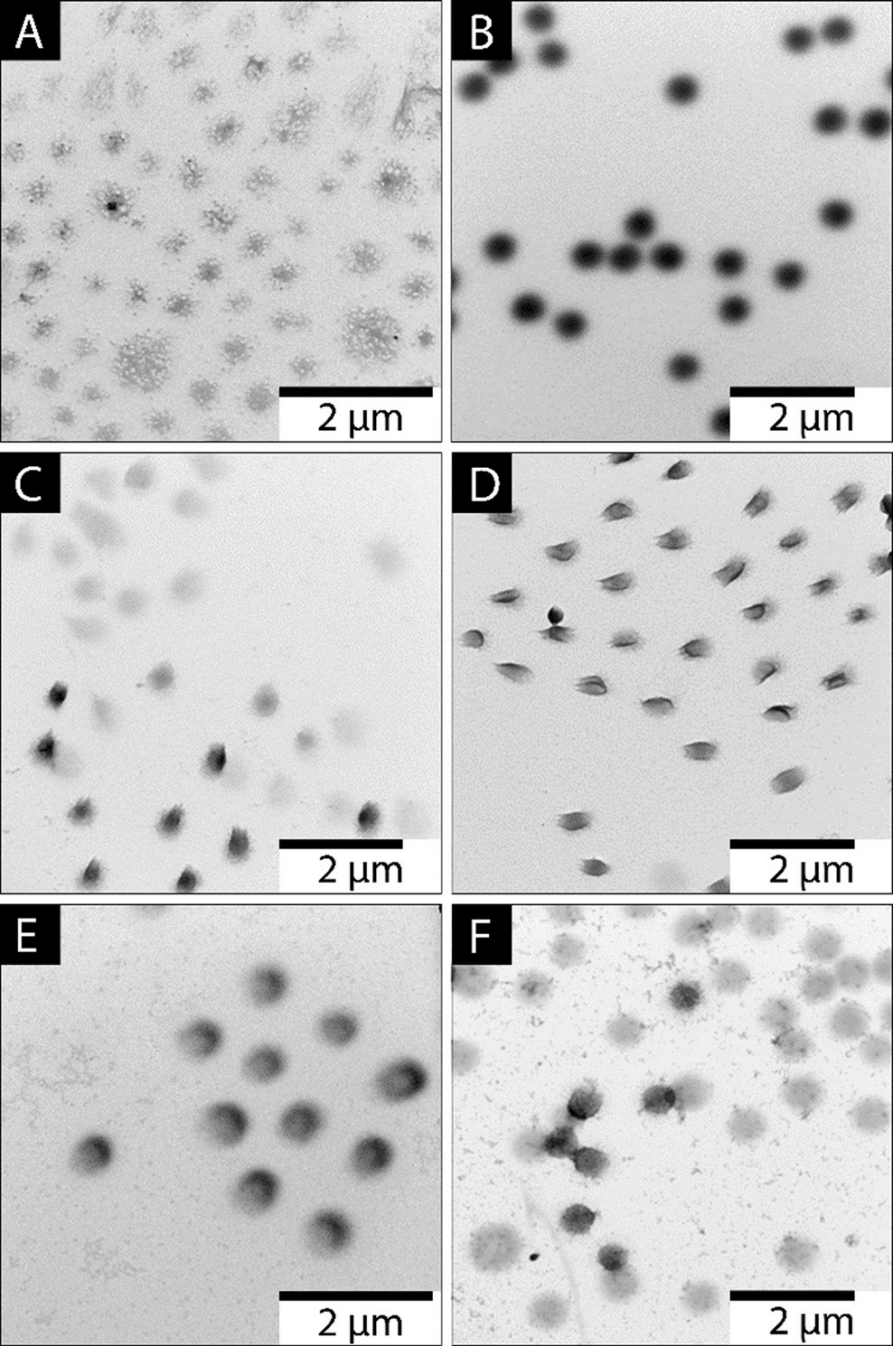
TEM images of NIPAm‐IA‐VIm microgels obtained (pH 6, 20 °C) after *t*_p_=10 min and *t*_mix_=0.5 min (A), 1 min (B), 2 min (C), 3 min (D), 4 min (E), and 5 min (F).

In order to optimize the formation of Janus‐like microgels, a second set of experiments was developed similar to the procedure described above. However, the polymerization time after mixing time was shortened to *t*_p_=10 min at 70 °C after mixing, to prevent the formation of an extra shell (Figure 2). The polymerization of the reaction mixture was stopped by rapid cooling and afterward purified by dialysis. All samples were first characterized by TEM, and the selected Janus‐like microgels (Figure 2 E) were further characterized by using different analytical techniques.

The reduction of the polymerization time *t*_p_ to 10 min resulted in the formation of Janus‐like microgels at *t*_mix_ between 2 min and 4 min (Figure 2 C–E). Similar to the set of reactions at *t*_p_=1 h (Supporting Information, Figure S2 A), microgels with a random distribution of charges were observed after 0.5 min and 1 min mixing time (Figure 2 A,B), since the mixing at this stage contains mostly unreacted monomers (Supporting Information, Figures S3 A, S4, S5, and S6). Starting from *t*_mix_=2 min, the formation of Janus‐like microgels took place (Figure 2 C–E). These TEM images visualize the evolution of the Janus‐like microgels, starting from the merging of two differently charged pre‐microgels to reaching the colloidal stable state of the Janus‐like microgels. The creation of stable Janus‐like microgels can be explained by coacervation process driven by the electrostatic attraction of the ionizable groups and the crosslinking effect that holds two pre‐ microgels together (Supporting Information, Figure S3 B). As expected, at *t*_mix_>4 min, microgels containing either VIm or IA were formed. According to the images, the darker microgels, corresponding to NIPAM‐IA microgels, are smaller than the NIPAM‐VIm microgels (Figure 2 F), which is also supported by the results of in situ DLS (Figure 1).

**Figure 3:**
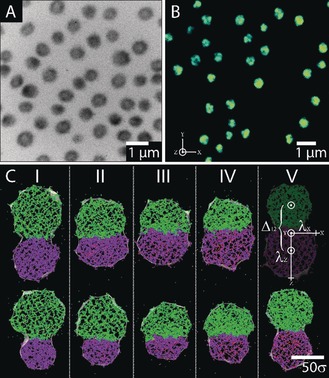
A) TEM image of NIPAm‐IA‐VIm Janus‐like microgels (*t*_p_=10 min; *t*_mix_=4 min) (pH 4, *T*=20 °C). B) Super‐resolved fluorescence microscopy image of NIPAm‐IA‐VIm Janus‐like microgels (*t*_p_=10 min; *t*_mix_=4 min) (pH 4, T=20 °C). C) Computer simulation snapshots of the symmetrical, Js, and asymmetrical, Ja, Janus‐like microgels formed by (top row) the same size precursor microgels, C_16k_(NIPAm‐VIm) + A_16_ (NIPAm‐IA), and (bottom row) by the different size precursor microgels, C_16k_(NIPAm‐VIm) + A_8k_(NIPAm‐IA) (Supporting Information, Table S4). Snapshots represent the slices of 10σ width of the equilibrated microgels through the center of mass of the microgel in the XZ plane. The main axis of the microgel coincides with the direction of the unit vector along the OZ axis and the center of coordinates match the center of mass of the microgel. NIPAm and VIm groups in the cationic precursor microgel, C, are marked in green and yellow, respectively. NIPAm and IA groups in the anionic precursor microgel, A, are marked in violet and pink, respectively. Symbols I, II, III, IV, and V denote the cases of different ratios of ionized groups IA:Vim within the microgel (emulation of the system at different pH values): I −0 %:10 %; II −2.5 %:7.5 %; III −5 %:5 %; IV −7.5 %:2.5 %; V −10 %:0 % (Supporting Information, Table S4).

As super‐resolved fluorescence microscopy (SFM)^23^ method, dSTORM^24^ has been proven to be very suitable for the imaging of microgels.^25^ It was also used here to study the morphology of the Janus‐like microgels in aqueous solution. Before measuring, Janus‐like microgels were labeled with a negatively charged fluorescent dye sodium 5,5′‐((perfluorocyclopent‐1‐ene‐1,2‐diyl)bis(2‐ethyl‐1,1‐dioxidobenzo[b]thiophene‐3,6‐diyl))bis(2‐methoxybenzenesulfonate)^26^ that effectively attaches to the imidazole group at pH 4 (Figure 3 B).

Figure 3 A reveals the morphology of Janus‐like microgels under acidic condition (pH 4), where protonation of imidazole groups take place, leading to a more prominent swelling of the NIPAm‐VIm based side. The SFM image in Figure 3 B confirms the same statement. As predicted, under the same operation environment (pH 4, 20 °C) but in the aqueous state, the microgels show a mushroom‐shaped structure where the labeled NIPAm‐VIm side is more pronounced than the NIPAm‐IA side which is in agreement with the TEM image (Figure 3 A).

**Figure 4:**
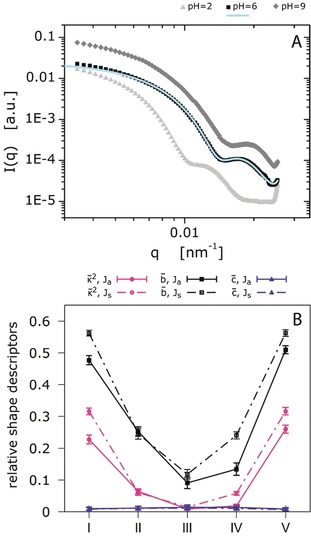
A) Particle form factors obtained via SLS using a laser wavelength of *λ*=640 nm. Experimental data are shown for pH 2, pH 6, and pH 9 at *T*=20 °C. The corresponding fit for pH 6 is based on the fuzzy sphere model. The data for pH 2 and pH 9 cannot be fitted based on spherical models. B) The dependence of sphericity, $\bar{b}$ , cylindricity, $\bar{c}$ , and relative shape anisotropy, $\bar{\kappa^{2}}$ of the symmetrical, Js, (solid lines) and asymmetrical, Ja, (dash‐dotted lines) Janus‐like microgels on the different ratio of ionized groups IA:VIm within the microgels: I −0 %:10 %; II −2.5 %:7.5 %; III −5 %:5 %; IV −7.5 %:2.5 %; V −10 %:0 %.

Polyampholyte microgels based on NIPAm are both temperature‐ and pH‐responsive.^27^ The microgel size measurements as a function of pH were performed with DLS to give a brief overview of how the microgels swell with various pH values (Supporting Information, Figure S7). The microgels are positively charged at pH<5.5 and negatively charged at pH>5.5. As a result, microgels are swollen both at lower pH 3 (R_H_=455±7 nm) and higher pH 10 (R_H_=431±8 nm). This discrepancy between the two swollen states can be explained with the help of Figure 4 A. The TEM image reveals that the unstained (VIm) part is slightly larger than the stained (IA) part. Therefore, protonation of the imidazole ring leads to a more prominent swelling. At the isoelectric point (pH 5.5), the microgels are in a collapsed state (R_H_=418±3 nm) due to the compensation of charges.

Static scattering methods are well known for allowing the investigation of polymer structures in solution.^28^ As discussed above, the uneven swelling of the different sides of the microgels is expected when changing the pH from 2, over 6 to 9. We used static light scattering (SLS) to distinguish between the different global structures of the microgels. We expect the fuzzy sphere model, which is commonly used for microgels, to match the particle form factor of the microgels at its isoelectric point (pH≈6), where the swelling is homogenous.^29^ Contrary, the fuzzy sphere model is expected to fail when fitting the particle form factors at pH 2 and pH 9. In these regimes, the microgels are most likely anisometric.

Figure 4 A shows the experimental data of the particle form factor for the three states of the microgel obtained via SLS. The data were fitted using the software *FitIt*!, and the implemented fuzzy sphere model.^22^ For pH 6 only one fit is shown. The fit not only matches the data but also shows physically reasonable characteristic parameters. Additional data on these microgels for a broader q‐range, as well as at high temperatures in their collapsed state are shown in Figures S13 and S14 in the Supporting Information. The fit results in a radius of R_SLS_=334±7 nm, while DLS^30^ gives R_H_=419±11 nm (Supporting Information, Figure S15). Since SLS is not sensitive to the dangling chains at the microgels periphery, the radius obtained via SLS is expected to be smaller than the hydrodynamic radius. At pH 6 and *T*=50 °C, the form factor can be fitted with the model of a homogeneous sphere.

However, the scattering data at pH 2 and pH 9 cannot be fitted with form factor models for spherical objects. The fuzzy sphere form factor model does not describe the experimental data (Supporting Information, Figure S13), which clearly demonstrates that the microgels are non‐spherical for pH values at which either one side is charged.

Further to the experimental results, the simulations of the equilibrium structure of the Janus‐like microgels in symmetric and asymmetric cases have been performed (Figure 3 C, top and bottom rows). In our designation, Janus‐like microgel is considered to be symmetric (Js) or asymmetric (Ja) when it is formed by the cationic and anionic precursor microgels of the same or different sizes (Supporting Information, Table S4). The quantities describing the shape and structure of the Js and Ja microgels were estimated for different pH values. Simulation on pH changes in the system was performed by a variation on the ratio of the ionized groups IA:VIm within the microgel (Supporting Information, Computer simulation (Model)). The average eigenvalues ${\bar{\lambda }}_{x}$ , ${\bar{\lambda }}_{y}$ , and ${\bar{\lambda }}_{z}$ (Supporting Information, Eq. (5)) after division by $R_{g}^{2}$ are plotted in Figure S9 in the Supporting Information. In all cases, microgels have an oblong shape, elongated in the direction of the major principal axis Z (Figure 3 C). The eigenvalues ${\bar{\lambda }}_{x}$ and ${\bar{\lambda }}_{y}{\bar{\lambda }}_{z}$ coincide with each other within the error, while ${\bar{\lambda }}_{z}>{\bar{\lambda }}_{x}$ , ${\bar{\lambda }}_{y}$ . The average relative asphericities $\bar{b}$ and the average relative acylindricities $\bar{c}$ (Supporting Information, Eq. (12)), as well as the average relative anisotropy coefficients ${\bar{\kappa }}^{2}$ (Supporting Information, Eq. (14)) are plotted in Figure 4 B. It is evident that at extreme pH points, I and V, where only cationogenic or anionogenic groups are ionized, microgels show very cylindrically symmetric shapes ($\bar{c}\approx 0$ , $\bar{b}$ around 0.5) and turn less cylindrical and more spherical ($\bar{c}\approx 0$ , $\bar{b}\approx 0.1$ ) near the isoelectric point (Figure 3 C, III (top) and IV (bottom)). According to the measurements of gyration radius $R_{g}$ (Supporting Information, Eq. (6)) as a function of pH, the microgel swells both at lower and higher pH values while it gradually shrinks when approaching the isoelectric point. The detailed examination of the swelling behavior based on the analysis of the distribution of beads within the microgel along the major principal axis, Z (Supporting Information, Figures S11 and S12) clearly indicates that at extreme pH points (I and V), the charged side of the Janus‐like microgel swells more, compared to the uncharged side. The reasons for this phenomenon are the electrostatic repulsion of similarly charged groups and the osmotic pressure of the counterion clouds. pH changes towards the isoelectric point lead to the appearance of pairs of oppositely charged subchains within the relevant parts of the microgels having a strong tendency to form neutral complexes. Directing attention at the peaks in Figure S11 (II–IV) in the Supporting Information, striking features become apparent: the complexation is accompanied by the collapse of the inner part of the Janus‐like microgel, redistribution of the masses within the microgel, increasing the contact area of oppositely charged precursor microgels, interpenetration of the subchains belonging to the different precursor microgels and release of the counterions. We expect these changes to become more pronounced for the case of a low crosslinked gel.

**Figure 5:**
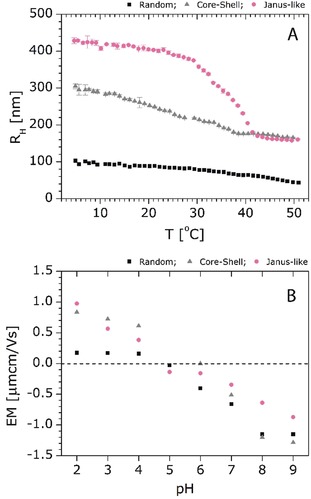
A) Hydrodynamic radii measured at different temperatures (pH 6) and B) electrophoretic mobility at different pH (*T*=20 °C) for NIPAm‐IA‐VIm microgels with random, core–shell, and Janus‐like distribution of charges. The dotted gray line in (B) at EM=0 is a guide to see the isoelectric point of the microgels better.

The thermo‐ and pH‐sensitivity of polyampholyte Janus‐like microgels and its difference from the polyampholyte microgels with random and core–shell distribution of ionizable groups are discussed in this section. Figure 5 A shows the temperature‐responsive behavior of all three polyampholyte microgels at pH 6, where the polyampholyte microgels should exhibit both positive and negative charges, leading to charge compensation. This is shown by the electrophoretic mobility in Figure 5 B. The swelling of polyampholyte microgels is influenced by two factors, I) the physical crosslinking within the opposite charged groups/subchains, and II) the counterions in the microgel network.6b, 31 Polyampholyte microgels with a random distribution of ionizable groups exhibit weak temperature responsiveness due to the efficient charge compensation, leading to more physical crosslinking and fewer counterions within the network. Interestingly, polyampholyte microgels with the same amounts of monomers but different distributions of VIm and IA groups behave completely differently upon temperature change.

Polyampholyte microgels with core–shell distribution of ionizable groups exhibit less temperature dependency and less swelling at lower temperatures compared to polyampholyte Janus‐like microgels. At pH 6, the oppositely charged groups are distributed in different compartments of the microgel. According to the type of microgels, the contact area (*A*) between the opposite charges is different (*A*_core‐shell_>*A*_Janus‐like_). Therefore, less physical crosslinking of the different ionizable groups and more counterions are present in the Janus‐like polyampholyte microgels, resulting in a more pronounced collapse when turning to a higher temperature. Another interesting observation is the shift of the volume phase transition temperature (VPTT, the temperature when the microgels turn from a highly swollen state to a collapsed state). While pure NIPAm microgels exhibit a VPTT at around 32 °C, the Janus‐like polyampholyte microgels show one at 35.9 °C. This effect can be attributed to the presence of the spatially separated hydrophilic monomer units (VIm and IA) in the microgels.6b Furthermore, the sharp temperature‐induced transition in the case of Janus‐like polyampholyte microgels can be explained by the reduction of the charge interference and absence of the “corset” effect reported for core–shell polyampholyte microgels.^31^

## Conclusion

In conclusion, we demonstrated a simple, and straightforward method for the synthesis of Janus‐like polyampholyte microgels. Our synthesis approach is based on the controlled coacervation and phase separation of growing polyelectrolyte microgel precursors during the precipitation polymerization process. Calorimetric studies, as well as in situ time‐dependent DLS measurements, were employed to identify the ideal mixing time (*t*_mix_) of two growing polyelectrolyte microgel populations. TEM and SFM images of polyampholyte microgels revealed that they have a Janus‐like distribution of ionizable groups. The synthesized polyampholyte Janus‐like microgels are both temperature‐ and pH‐sensitive. The pH‐sensitivity enables particles to be swollen at low and high pH while being collapsed at the isoelectric point.

The synthesized polyampholyte Janus‐like microgels are unique colloids with a broad range of useful applications. They could potentially be used as novel drug carriers for simultaneous delivery and controlled, on‐demand release of different drugs. Polyampholyte Janus‐like microgels can act as compartmentalized catalyst carriers for the combination of enzymes and organocatalysts or metal complexes in tandem reactions.

## Conflict of interest

The authors declare no conflict of interest.

## Supporting information

As a service to our authors and readers, this journal provides supporting information supplied by the authors. Such materials are peer reviewed and may be re‐organized for online delivery, but are not copy‐edited or typeset. Technical support issues arising from supporting information (other than missing files) should be addressed to the authors.

Supplementary

Supplementary

Supplementary
