# Supplementary material for: Synthesis of Polyampholyte Janus‐like Microgels by Coacervation of Reactive Precursors in Precipitation Polymerization
Source: Angew Chem Int Ed Engl. 2019 Dec 10;59(3):1248–55. doi: 10.1002/anie.201910450 (PMC6973257; doi:10.1002/anie.201910450)
Supplement: Supplementary file 1 — Supplementary [file ANIE-59-1248-s001.pdf]

Angewandte Chemie
Eine Zeitschrift der Gesellschaft Deutscher Chemiker

Supporting Information

Synthesis of Polyampholyte Janus-like Microgels by Coacervation of Reactive Precursors in Precipitation Polymerization

*Wenjing Xu, Andrey Rudov, Alex Oppermann, Sarah Wypysek, Michael Kather, Ricarda Schroeder, Walter Richtering, Igor I. Potemkin, Dominik Wöll, and Andrij Pich**

anie_201910450_sm_miscellaneous_information.pdf
anie_201910450_sm_video1_s.mp4
anie_201910450_sm_video2_s.mp4

Supporting Information

Table of Contents

Table S1: Reagents used for the synthesis of polyampholyte Janus-like microgels. 2
Table S2: Reagents used for the synthesis of polyampholyte microgels with a random distribution of ionizable groups. 2
Table S3: Reagents used for the synthesis of polyampholyte core-shell microgels. 3
Table S4: Types and amount of beads used in the simulation synthesis of polyampholyte Janus-like microgels. 4
Table S5: Types and amount of beads used in the simulation synthesis of polycationic and polyanionic pre-microgels. 4

Figure S 1: Calorimetric measurements for pure NIPAm microgels (black) and NIPAm-VIm microgels (red). The numbers give the total reaction heat. 7
Figure S 2: TEM images of NIPAm-IA-VIm microgels obtained after t_p = 1 h and t_{mix} = (a) 1 min; (b) and (c) 3 min; (d) 5 min. 8
Figure S 3: The formation procedure of opposite charged precursor particles/microgels to different type of charged microgels. A) formation of mixed monomers to polyampholyte microgels with random dirstribution of ionizable groups. B) formation of pre-microgels into stable polyampholyte Janus-like polyampholyte microgels. C) formation of differently charged polyelectrolyte microgels. 9
Figure S 4: Computer simulation: formation of microgels with a random distribution of ionizable groups from the mixture of polycationic and polyanionic pre-microgel nuclei. Case of short mixing time (System 1). The middle row shows only a few pre-microgel nuclei of the systems that, over time, form into large clusters. The top row shows the center of masses of the microgels plotted in the middle row. Colored microgel nuclei are polyanionic, while grayish microgel nuclei are polycationic. The number of cationic and anionic microgel nuclei before mixing are equal ($NC_{0.5k}$ = $NA_{0.5k}$ = 32). Blue dots correspond to the counterions. 10
Figure S 5: Computer simulation: the fraction of clusters formed by the electrostatic interaction of oppositely charged pre-microgel nuclei as a function of time. Value 1.0 corresponds to the initial state of the system where all clusters consist of 1 microgel nucleus. The value 0.5 means that the number of clusters has halved due to its sticking. The number of cationic and anionic microgel nuclei before mixing are equal NC_{2k} = NA_{2k} = 8 (black circles), NC_{1k} = NA_{1k} = 16 (red triangles) and NC_{2k} = NA_{2k} = 8 (blue diamonds). 11
Figure S 6: Computer simulation: example of a quartet - the cluster formed by 4 oppositely charged pre-synthesized microgel nuclei. The number of cationic and anionic microgel inside the cluster is equal 2. Colored microgel nuclei are polyanionic, while grayish microgel nuclei are polycationic. 12
Figure S 7: Hydrodynamic radius R_H as a function of pH of Janus-like microgels at 20 °C. 12
Figure S 8: TEM images of the morphology of polyampholyte microgels with (A) random, (B) core-shell, and (C) Janus-like distribution. 13
Figure S 9: Three eigenvalues of the average instantaneous radius of gyration tensor, divided by the mean-square radii of gyration, vs. the different ratio of ionized groups IA:VIm within the microgels: I – 0%:10%; II – 2.5%:7.5%; III – 5%:5%; IV – 7.5%:2.5%; V – 10%:0%. Vertical bars denote standard deviations of the mean values. Cases of (A) Symmetric and (B) Asymmetric Janus-like microgels. 13
Figure S 10: (A) Average radius of gyration and (B) distance between the centers of mass of precursor cores within the Janus-like microgel as the functions of the different ratio of ionized groups IA:Vim within the microgels: I – 0%:10%; II – 2.5%:7.5%; III – 5%:5%; IV – 7.5%:2.5%; V – 10%:0%. Vertical bars denote standard deviations of the mean values. 14
Figure S 11: The fraction of beads of symmetric Janus-like microgels, Js (with respect to the total number of beads in the microgel) in a slice thickness of 1σ as a function of Z coordinate in its principal axis system. I, II, III, IV, and V denote the different ratio of ionized groups IA:VIm within the microgels: I – 0%:10%; II – 2.5%:7.5%; III – 5%:5%; IV – 7.5%:2.5%; V – 10%:0%. Vertical bars denote standard deviations of the mean values. 14
Figure S 12: The fraction of beads of asymmetric Janus-like microgels, Ja (with respect to the total number of beads in the microgel) in a slice thickness of 1σ as a function of Z coordinate in its principal axis system. I, II, III, IV, and V denote the different ratio of ionized groups IA:VIm within the microgels: I – 0%:10%; II – 2.5%:7.5%; III – 5%:5%; IV – 7.5%:2.5%; V – 10%:0%. Vertical bars denote standard deviations of the mean values. 15
Figure S 13: Particle form factors obtained via SLS using a laser wavelength of λ = 407 nm (left) and λ = 640 nm (right). Experimental data are shown for pH = 2, pH = 6 and pH = 9 at T = 20 °C. The corresponding fits are based on the fuzzy sphere model and are either matching the physical parameters or the experimental data (pH = 2 and 9), or both (pH = 6). 16

Figure S 14: Particle form factors obtained via SLS using a laser wavelength of λ = 407 nm (left) and λ = 640 nm (right). Experimental data are shown for pH = 6 in the swollen state at T = 20 °C and the collapsed state at T = 50 °C. The corresponding fits are based on the fuzzy sphere model........16
Figure S 15: Plot of the decay rate of a second order cumulant fit against q^2 for the microgels at pH = 2, 6 and 9 and T = 20 °C (left) and pH = 6 at T = 20 °C and T = 50 °C (right). 17

Experimental Part:

Reagents and Materials: *N*-Isoprylacrylamide (NIPAm, 97%), Itaconic acid (IA, ≥ 99%), 1-Vinylimidazole (VIm, ≥ 99%), 2,2'-Azobis[2-methylpropionamidine] dihydrochloride (AMPA, granular, 97%), and *N,N*'-Methylene(bis)acrylamide (BIS, 99%) were purchased from Sigma Aldrich. NIPAm was recrystallized from hexane and dried under vacuum before use. VIm was cleaned by running through an Al_2O_3 column. Water used in the experiments was purified using a Millipore water purification system with a minimum resistivity of 18 MΩ*cm.

Synthesis of polyampholyte Janus-like microgels. For the synthesis of Janus-like microgels, two separate reactions were carried out in two different reaction vessels (vessel 1: NIPAm-VIm, vessel 2: NIPAm-IA).

Synthesis 1. NIPAm, VIm, and BIS were added in 100 mL aqueous buffer (pH = 3). After heating up to 70 °C, the solution was stirred at 200 rpm under nitrogen atmosphere for 1 h.

Synthesis 2. NIPAm, IA, and BIS were added in 100 mL aqueous buffer (pH = 10). After heating up to 70 °C, the solution was stirred at 200 rpm under nitrogen atmosphere for 1 h.
The initiator AMPA was added to synthesis 2 two min after synthesis 1 since NIPAm-VIm reacts faster than NIPAm-IA. 1 mL of each solution was removed after 0.5, 1, 2, 3, 4, and 5 min. The two solutions were immediately combined in one pre-heated flask and allowed to stir for 1 h (first set of experiments) or 10 min (second set of experiments) at 70 °C at 200 rpm. All microgels were purified directly after the polymerization using a composite regenerated cellulose membrane from Millipore (NMWCO 12,000 – 14,000) for 3 d.

Table S1: Reagents used for the synthesis of polyampholyte Janus-like microgels.

Synthesis	NIPAm		VIm		IA		BIS		AMPA		Remark
	g	mmol	g	mmol	g	mmol	g	mmol	g	mmol	ratio: NIPAM.VIm/IA
Synthesis 1	1	8.837	0.166	1.764			0.05	0.324	0.038	0.140	80:20
Synthesis 2	1	8.837			0.287	2.206	0.05	0.324	0.038	0.140	80:20

Synthesis of reference polyampholyte microgels with a random distribution of ionizable groups. Microgels with a random distribution of ionizable groups were synthesized via one-step free-radical precipitation polymerization.[1] All monomers (NIPAm, VIm, IA) and cross-linker (BIS), the appropriate amount was first mixed in 80 mL distilled water under N_2 atmosphere at 70 °C in a double-wall reactor under constant stirring for 1 h. The addition of the initiator AMPA afterward started the reaction and allowed to polymerize for 4 h. The synthesized microgel solution was then directly cleaned via dialysis using a composite regenerated cellulose membrane from Millipore (NMWCO 12,000-14,000) for 5 d.

Table S2: Reagents used for the synthesis of polyampholyte microgels with a random distribution of ionizable groups.

Synthesis	NIPAm		VIm		IA		BIS		AMPA		Remark
	g	mmol	g	mmol	g	mmol	g	mmol	g	mmol	ratio: NIPAM.VIm:IA
Random	0.650	5.744	0.135	1.436	0.187	1.436	0.035	0.230	0.035	0.129	60:20:20

Synthesis of reference polyampholyte microgels with core-shell distribution of ionizable groups. Microgels with a core-shell distribution of ionizable groups were synthesized in a two-step approach employing free-radical precipitation polymerization in aqueous media wherein the first step core microgels were synthesized followed by the addition of the shell in the second step.[1-2] For the core-microgel, appropriate amounts of NIPAm, IA, and BIS were dissolved in 150 mL distilled water and heated up to 70 °C while purging with N_2 in a stirred round-bottom flask. After 1 h, the initiator AMPA was added, and the reaction was carried out for 2 h under constant stirring. After the synthesis, the core microgels were remained in the round-bottom flask without cooling down or cleaning. Subsequently,

the appropriate amounts of NIPAm, VIm, BIS; and AMPA for the shell synthesis were dissolved in pre-heated distilled water (70 °C, 150 ml) and quickly added to the core dispersion. The reaction was again allowed to continue at 70 °C for a further 2 h. The core-shell microgel dispersions were cleaned by dialyzing for 3 d against water using a composite regenerated cellulose membrane from Millipore (NMWCO 12,000-14,000).

Table S3: Reagents used for the synthesis of polyampholyte core-shell microgels.

Synthesis	NIPAm		VIm		IA		BIS		AMPA		Remark
	g	mmol	g	mmol	g	mmol	g	mmol	g	mmol	ratio: NIPAM.VIm/IA
Core	1.697	14.837	-	-	0.488	3.751	0.087	0.564	0.102	0.376	80:20
Shell	1.697	14.837	0.355	3.772	-	-	0.087	0.564	0.102	0.376	80:20

Computer Simulation (Model). Brownian molecular dynamics (MD) simulations within a standard coarse-grained model with implicit solvent were performed at the supercomputer JURECA, Jülich Supercomputing Centre. The LAMMPS package was used.[3] Without loss of generality, the simulations were carried out based on dimensionless units, where the fundamental quantities such as mass *m*, the diameter of the bead σ, and the Boltzmann constant k_B are considered to be equal to 1. All structural units of the microgels (charged and uncharged monomer units, cross-linkers) and counterions are modeled as Lennard-Jones particles (beads) of the same diameter and mass. Electrostatic interactions between any pair of charged particles are described by Coulomb potential. P^3M algorithm with the accuracy of 10^{-5} has been used. The calculations were carried out in the NVT ensemble with periodic boundary conditions, which is quite efficient for models with implicit solvent.[4] The equations of motion were integrated with a time step Δt = 0.005τ, where τ is the standard time unit for a Lennard-Jones fluid.

In the experiment, precipitation polymerization was used to obtain Janus-like microgels from the pre-synthesized microgel nuclei, i.e., precursor particles. In this approach, a mixture of reagents, growing polymeric chains and nuclei of both types dissolved in water heated up to 70 °C is polymerized to produce microgel particles. Gelation and formation of particles of complex morphologies are achieved both by transfer reactions and by the presence of multifunctional monomer. We did not aim to reproduce the described processes of Janus-like microgel formation in details in a computer simulation. However, we believe that initial nuclei characteristics, including sizes, gelation state, cross-linking degree, distribution of active sites, among other things, are crucial for the final Janus-like microgel structure. We introduce the simplified scheme of preparation of Janus-like microgel with pre-defined morphology used in our study. At the first stage, we prepared cationic, C, and anionic, A, microgel precursors (Table S4). It was shown in the experiment that the presence of co-monomers IA slows down the polymerization rate of NIPAm-IA (Figure 1) microgels in comprising to the NIPAm-VIm microgels. 2 min delay between initiation of NIPAm-IA and NIPAm-VIm microgels were used to start the synthesis of Janus-like microgels from the comparable size precursors. However, the gap between the sizes of growing oppositely charged microgel precursors before mixing increases over time. Thus depending on mixing time, one can expect to receive both symmetric and asymmetric Janus-like particles. Based on these speculations, we prepared several microgel precursors with different molecular weight: small (A_{8k} containing 8006 beads) and two large ones (A_{16k} and C_{16k} containing 16135 beads). We will explore how the molecular weight of one of the components of Janus-like microgel influences on their morphology and internal structure.

For this purpose, we initially constructed neutral microgels in a similar way as reported in ref.1. Microgels consisted of fully stretched chains of equal length of n = 10 neutral beads (O), which are connected through tetra functional cross-linker beads (X). The fraction of the cross-linkers for such microgels is around 0.05. The connectivity of the beads into a polymer network was maintained by a combination of the finite extension nonlinear elastic (FENE) potential and Lennard-Jones potential:[5]

$$U_{bond}(r) = U_{FENE}(r) + U_{LJ}(r) \quad (1)$$

where the distance between the two beads is denoted by *r*.

$$U_{FENE}(r) = -\frac{1}{2} K {R_{max}}^2 \ln(1 - \frac{r^2}{{R_{max}}^2}) \quad (2)$$

$$U_{LJ}(r) = 4\varepsilon_{bond} \left[\left(\frac{\sigma}{r}\right)^{12} - \left(\frac{\sigma}{r}\right)^{6} \right] + \varepsilon_{bond} \quad (3)$$

with spring constant, $K = 30k_BT/\sigma^2$, maximum bond length, $R_{max} = 1.5\sigma$, $\varepsilon_{bond} = 1k_BT$, and cutoff radius $r_{cut} = 2^{1/6}\sigma$.

The interactions between any pair of the beads were described through the truncated-shifted Lennard-Jones potential.[6] To mimic the reaction temperature of 70 °C (which was suitable in the experiment in terms of polymerization rates and microgel yield) we set the value of the Lennard-Jones parameter for microgel bead to bead interactions, $\varepsilon_{b\text{-}b} = 1k_BT$ (b ∈{O, X, I^+, I^-, R_C, R_A}) and cutoff distance r_{cut}= 2.5σ. These values of the parameters correspond to the case of a bad solvent.

Then ionizable groups were introduced. At pH = 10 $\varphi^-_{max} = 10\%$ of microgel beads (O) in the A_{8k} and A_{16k} microgel were randomly selected and converted into negatively charged beads (I^-). By analogy, at pH = 2, $\varphi^+_{max} = 10\%$ of neutral beads (O) in C_{16k} microgels has been modified to the positively charged beads (I^+). To mimic the effect of pH changes, the fractions of charged beads φ^- and φ^+ were considered to be linearly dependent on pH value (see ref 1 for details). For example, at pH = 6, C_{16k} and A_{16k} contains $\varphi^+ = \varphi^- = 5\%$ of charged groups. At each pH value, the necessary amount of positively and negatively charged counterions are added into the simulation boxes to provide overall electric neutrality. The value of the Lennard-Jones parameter and cutoff distance for microgel bead-counterion and counterion-counterion interactions were set to $1k_BT$ and $2^{1/6}\sigma$ respectively. The C_{16k}, A_{8k} and A_{16k} microgels with a distribution of charged groups corresponding to pH = 6 were placed into separated cubic simulation boxes of the volume $V = 200^3\ \sigma^3$ and pre-equilibrated during the $10 \cdot 10^6$ time steps.

After the equilibration, reactive sites were defined. 10% of neutral beads (O) of microgels were randomly selected and converted into reactive beads. Reactive beads of cationic, C, and anionic, A, microgel precursors are denoted as R_C and R_A, respectively. During the simulation runs, these groups could create new bonds according to some specified criteria. If two beads I and J of microgels are within a distance less than 1.5σ of each other, and if I is of bead type Rc and J is of bead type R_A, and if a bond does not already exist between I and J beads then with probability p_{bond} = 50% new bond pair is created. Thereafter, we prepared two cubic simulation boxes of the volume $V = 2{\cdot}200^3\ \sigma^3$. In one of them, we placed a pair of modified C_{16k} / A_{8k} microgels with counterions, and in the other one - C_{16k} / A_{16k} microgels with counterions. The interaction parameters in the system were identical to the ones described above. The distance between the centers of mass of the microgels was chosen to be equal 40σ ensuring the absence of direct contact. We assign initial speeds to the microgels 1 σ/τ in the direction towards each other and start the simulation run. During the collision of the microgels, we monitor the emergence of new bonds. Check for possible new bonds is performed every $t_{bond} = 10^3$ time steps during $t_{run} = 10^6$ time steps. In our scheme contact area and degree of interpenetration of the microgel precursors (with a fixed crosslinker density and fraction of charged groups per microgel) is mainly determined by the number of reactive sites and combination of p_{bond} and t_{bond} values. The higher value provides more crosslinked Janus-like microgel.

After fixing the primary structures of the Janus-like microgels, we investigated their internal structure, shape, and morphology at different values of pH in a good solvent, $\varepsilon_{b\text{-}b} = 0.01k_BT$ (b ∈{O, X, I^+, I^-, R_C, R_A}). The calculations were carried out in an NVT ensemble. The simulation last $10{\cdot}10^6$ steps, which is enough to approach the equilibrium states of the systems.

Table S4: Types and amount of beads used in the simulation synthesis of polyampholyte Janus-like microgels.

Type	Bead	Description	Amount	Fraction
	O	Neutral monomers	12835	80%
C_{16k}	X	Cross-linkers	800	5%
16035 beads	I^+	Ionizable groups	1600	10%
	R_C	Reactive sites	800	5%
	O	Neutral monomers	12835	80%
A_{16k}	X	Cross-linkers	800	5%
16035 beads	I^-	Ionizable groups	1600	10%
	R_A	Reactive sites	800	5%
	O	Neutral monomers	6406	80%
A_{8k}	X	Cross-linkers	400	5%
8006 beads	I^-	Ionizable groups	800	10%
	R_A	Reactive sites	400	5%

Table S5 shows additional simulations to support the following conclusions: a) short mixing time leads to the formation of polyampholyte microgels with random distribution of ionizable groups, and b) the formation of the polyampholyte microgels with random distribution of ionizable groups are also electrostatic driven.

For this, we prepared a series of small cationic, $C_{0.5k}$, C_{1k}, C_{2k}, and anionic $A_{0.5k}$, A_{1k}, A_{2k} pre-microgel nuclei (Table A 1) containing 10% of charged groups and mixed them with each other in a cubic simulation boxes of $V = 170^3\ \sigma^3$ with equal proportions: System 1: $NC_{0.5k} = NA_{0.5k} = 32$, System 2: $NC_{1k} = NA_{1k} = 16$ and System 3: $NC_{2k} = NA_{2k} = 8$. The total amount of beads in the systems are the same, and equal to the total amount of the beads unsed for the Janus-like microgel, $C_{8k}A_{8k}$ (Supporting Information Table S4). For system 1 with the shortest mixing time, the nuclei consist of several polyelectrolytes which are slightly crosslinked to each other. The higher the amount of beads per nuclei, the longer the mixing time we model. To mimic the temperature of 70 °C we have set the value of the Lennard-Jones parameter for the microgel bead to bead interactions to $\varepsilon_{b\text{-}b} = 1k_BT$ (b ∈ {O, X, I^+, I^-}) and the cutoff distance to r_{cut}= 2.5σ. In order to simplify the system, we didn`t neclected several aspects (without diminishing the importance of each of them): the chemical structure of the monomers, the presence of low molecular reagents in the system, growth of the nuclei and the polymeric chains over the time.

The calculations were carried out in a NVT ensemble. The systems were equilibrated $20{\cdot}10^6$ simulation steps. To increase the diffusion rate of the microgels the mass of each bead were reduced 100 times.

Table S5: Types and amount of beads used in the simulation synthesis of polycationic and polyanionic pre-microgels.

System 1	$NC_{0.5k} = NA_{0.5k} = 32$			
	Bead	Description	Amount	Fraction
$C_{0.5k}$	O	Neutral monomers	431	85%
506 beads	X	Cross-linkers	25	5%
	I^+	Ionizable groups	50	10%
$A_{0.5k}$	Bead	Description	Amount	Fraction

506 beads	O	Neutral monomers	431	85%
	X	Cross-linkers	25	5%
	I^-	Ionizable groups	50	10%
System 2	$NC_{1k} = NA_{1k} = 16$			
	Bead	Description	Amount	Fraction
C_{1k}	O	Neutral monomers	862	85%
1012 beads	X	Cross-linkers	50	5%
	I^+	Ionizable groups	100	10%
	Bead	Description	Amount	Fraction
A_{1k}	O	Neutral monomers	862	85%
1012 beads	X	Cross-linkers	50	5%
	I^-	Ionizable groups	100	10%
System 3	$NC_{2k} = NA_{2k} = 8$			
	Bead	Description	Amount	Fraction
C_{2k}	O	Neutral monomers	1724	85%
2024 beads	X	Cross-linkers	100	5%
	I^+	Ionizable groups	200	10%
	Bead	Description	Amount	Fraction
A_{2k}	O	Neutral monomers	1724	85%
2024 beads	X	Cross-linkers	100	5%
	I^-	Ionizable groups	200	10%

Calorimetric studies*.* Calorimetric measurements were performed in a reaction calorimeter RC1e from Mettler Toledo with a 500 mL 3-wall AP01-0.5-RTCal reactor equipped with Hastelloy® stirrer, a baffle, and a TurbidoTM turbidity probe from Solvias. The measurements were done in isothermal mode, in which the desired reaction temperature (Tr) is set at a constant value, and the jacket temperature (Tj) changes automatically to maintain Tr at the desired value. The amounts of monomer used can be seen in Table S1 (for 100 mL). 300 mL of the aqueous solution was heated up to 70 °C under a nitrogen atmosphere and constant stirring at 200 rpm. After 30 min, the respective amounts of monomers were added. Simultaneously, a probe recorded the heat generated. Once a stable baseline in the produced heat was reached (the minimal waiting time was 30 min), the initiator AMPA was added. The production of heat and the turbidity were recorded *in-situ* during the reaction using the iControl RCLeTM 5.0 software. The reaction was allowed to continue for 3 h and subsequently cooled down to room temperature. For the determination of the net reaction time, the end of the reaction was defined to be when the heat flow was back to a value close to zero and constant. The fractional conversion can be obtained by integrating the reaction heat curve.
Static Light Scattering*.* Experimental data of the particle form factors of the microgels were obtained via static light scattering. All experiments were performed on a closed goniometer by SLS-Systemtechnik GmbH, equipped with lasers of three different wavelengths, namely 407, 640, and 819 nm. Sample preparation was achieved via filtration using 0.8 µm cellulose acetate filters by *Sartorius*. The solutions were highly diluted to minimize multiple scattering and contributions to the structure factor. Scattering angles were varied between 15° and 150° in 1° steps. By using a wavelength of λ = 407 nm a q-range of 0.007 nm^{-1} to 0.04 nm^{-1} is covered, while λ = 640 nm covers a q-range of 0.003 nm^{-1} to 0.025 nm^{-1}. The temperature was controlled by a thermostat (Julabo CF40) connected to the goniometer and set to 20 °C or 50 °C, respectively. The solvent intensity was measured and subtracted as background. Scattering intensity was measured and imported to FitIt!.[7] Particle form factors were fitted using the implemented fuzzy sphere model. Back-reflection of the glass cuvettes was corrected for all measurements.
Dynamic Light Scattering and Electrophoresis*.* Dynamic light scattering (DLS) was used to obtain reference values for the microgel sizes. The same samples as used for SLS were used for DLS. A setup of an ALV goniometer and a HeNe laser of λ = 633 nm was used. The system was equipped with a digital hardware correlator and two avalanche photodiodes. The intensity was measured for 90 s at angles varied between 30° and 52° in 2° steps. The temperature was adjusted to 20 °C or 50 °C using a cryostat and an index-match-bath filled with toluene. The decay rate Γ from a second-order cumulant fit was plotted against q^2. The diffusion coefficient was calculated via linear regression of this plot ($\Gamma = q^2 D_0$).[8] The hydrodynamic radii were calculated based on the Stokes-Einstein equation and used as a reference for appropriate values for the radius for the particle form factor fits.[9]
The electrophoretic mobility and the diffusion coefficient of the microgel particles were measured using a Zetasizer NanoZS (Malvern, UK). Measurements were taken at 20 °C after equilibrating the samples for at least 15 min. pH trends were measured from 2 to 12 in 1 steps using self-prepared buffer systems where the ionic strength is kept at 30 mM if no other additional information provided.
T-trends were measured from 5 °C to 50 °C in 1 °C steps using an ALV/LSE 7004 Tau Digital Correlator and a JDS Uniphase laser operating at 633 nm. Before all measurements, the samples were filtered with a 1.2 µm PET-filter. The hydrodynamic radius R_H can be obtained from the diffusion coefficient by the Einstein-Stokes equation.
Transmission Electron Microscopy*.* To visualize the distribution of functional groups within the microgel, transmission electron microscopy (TEM) was performed with a Zeiss Libra TM 120 (Carl Zeiss, Oberkochen, Germany). 20 µL of each sample was stained with 5 µL of uranyl acetate $U(Ac)_3$, and one drop was put on a carbon-coated copper grid. Samples were then dried overnight.
Super resolved fluorescence microscopy*.* Super-resolved fluorescence microscopy images were taken with a custom-built microscopy setup. The beam of a 488 nm diode laser (Cobolt Jive, 200 mW) was extended using a reflective optical beam expander (BE06R, Thorlabs) and focused to the back focal plane of a 100x/1.3 NA oil immersion objective (UPLFLN100xO2, Olympus) mounted

in an Olympus IX83 inverted microscope. The fluorescence was collected using the same objective and was spectrally separated from the excitation laser light by a quad-line beamsplitter (zt405/488/561/640rcp, AHF Analysentechnik Tübingen). The image was further magnified by two lenses (f1 = 200 mm AC245-200-A and f2 =400 mm AC254-400-A, Thorlabs) and imaged onto the chip of an EMCCD camera (Andox iXON Ultra 897). To eliminate the background from the residual laser light, an additional bandpass filter (525/30 BrightLine HC, AHF Analysentechnik Tübingen) was placed between the two magnifying lenses. The recorded data were analyzed using the ImageJ plugin ThunderSTORM[10] and visualized using the software ViSP[11].
The positive VIm containing part of the microgels was labelled by addition of the negatively charged fluorescent dye sodium 5,5'-((perfluorocyclopent-1-ene-1,2-diyl)bis(2-ethyl-1,1-dioxidobenzo[b]thio-phene-3,6-diyl))bis(2-methoxybenzenesulfonate)[12] to the microgel dispersion at pH 4 and stirring overnight. To remove the non-bound dye, the dispersion was dialyzed against pH 4 buffer. The microgels were spin-coated onto a cleaned coverslip, and a drop of buffer was added to rehydrate the microgels.

Computer Simulation (Characterization). The geometry of Janus-like microgels such as size, aspect ratio, form, and shape play a significant role in their particle orientation, surface activity, and packing geometry in bulk and at an interface. In our study, we calculated various shape descriptors derived from the gyration tensor, which would describe the geometry of the microgel.
We define the gyration tensor as

$$\mathbf{S} = \frac{1}{N}\sum_{i=1}^{N} \boldsymbol{s}_i \boldsymbol{s}_i^T = \overline{\boldsymbol{s}\boldsymbol{s}^T} = \begin{bmatrix} \overline{x^2} & \overline{xy} & \overline{xz} \\ \overline{yx} & \overline{y^2} & \overline{yz} \\ \overline{zx} & \overline{zy} & \overline{z^2} \end{bmatrix}, \quad (4)$$

where $\boldsymbol{s}_i = \begin{bmatrix} x_i \\ y_i \\ z_i \end{bmatrix}$ is the position vector of each bead, which is considered with respect to the center of mass of the microgel $\sum_{i=1}^{N} \boldsymbol{s}_i = 0$, and the overbars denote an average over all beads N in the microgels. To get a complete characterization of the Janus-like microgels, we computed the eigenvalues of the gyration tensor $\lambda_x = \overline{X^2}, \lambda_y = \overline{Y^2}, \lambda_z = \overline{Z^2}$. The gyration tensor is symmetric thus, the Cartesian coordinate system can be found in which it is diagonal. Transformation to the principal axis system diagonalizes S, and we choose that principal axis system in which

$$\mathbf{S} = \mathrm{diag}(\lambda_x, \lambda_y, \lambda_z), \quad (5)$$

where we assume that the eigenvalues of ***S*** are sorted in ascending order, i.e., $\lambda_x \leq \lambda_y \leq \lambda_z$ (Figure 3C V) and x, y, and z are the new coordinate axes. These eigenvalues are called the principal moments of the gyration tensor. The first invariant of S gives the squared radius of gyration,

$$\mathrm{tr}\,(\boldsymbol{S}) \equiv I_1 = \lambda_x + \lambda_y + \lambda_z = R_g^2, \quad (6)$$

a measure of the average size of the particular conformation. Microgel consisting of beads of equal mass have by definition a moment of inertia tensor (with respect to the center of gravity), **I**, which is diagonal in the same frame of reference as **S**.
By analogy with ref.[13], we define the shape anisotropy of microgel in a particular conformation as the traceless deviatoric part of **S**

$$\hat{\mathbf{S}} = \mathbf{S} - \frac{1}{3}\mathrm{tr}\,(\mathbf{S})\mathbf{E}, \quad (7)$$

$\mathrm{tr}\,(\hat{\mathbf{S}}) = 0$ where **E** is the unit tensor. In the principal axis system $\hat{\boldsymbol{S}}$, could be presented by a sum of two terms (in a way similar to molecular polarizability tensor)[14]

$$\hat{\mathbf{S}} = B\,\mathrm{diag}(^2/_3, -^1/_3, -^1/_3) + C\,\mathrm{diag}(0, ^1/_2, -^1/_2), \quad (8)$$

where we define quantities such as the asphericity, *B,* and the acylindricity, *C*, of a Janus-like microgel, derived from the values of the principal moments

$$B = \lambda_z - \frac{1}{2}(\lambda_x + \lambda_y), \quad (9)$$

$$C = \lambda_y - \lambda_x, \quad (10)$$

which are always not negative. When the bead distribution within the microgel is spherically symmetric or has a cubic, a tetrahedral or higher symmetry, then the three principal moments are equal, λx = λy = λz and b = 0. The acylindricity is zero only when the two principal moments are equal, λx = λy. This zero condition is met when the distribution of particles is cylindrically symmetric and whenever the particle distribution is symmetric with respect to the two coordinate axes. In our study, we use dimensionless relative quantities which were taken with respect to I_1

$$b = \frac{1}{\mathrm{Tr\,S}}B, \qquad 0 \leq b \leq 1 \quad (11)$$

$$c = \frac{1}{\mathrm{Tr\,S}}C, \qquad 0 \leq c \leq 1 \quad (12)$$

An overall measure of shape anisotropy is the quantity of $\mathrm{tr}\,(\hat{\boldsymbol{S}}\hat{\boldsymbol{S}})$,

$$\mathrm{tr}\,(\hat{\boldsymbol{S}}\hat{\boldsymbol{S}}) = \frac{2}{3}B^2 + \frac{1}{2}C^2 = \frac{2}{3}{I_1}^2 - 2I_2, \quad (13)$$

where $I_2 = \lambda_x\lambda_y + \lambda_x\lambda_z + \lambda_y\lambda_z$ is the second invariant of **S**. The dimensionless relative shape anisotropy κ^2 is defined as

$$\kappa^2 = \frac{3}{2}\,\mathrm{tr}\frac{(\hat{S}\hat{S})}{\mathrm{tr}\,(\boldsymbol{S})^2} = 1 - \frac{3\,I_2}{{I_1}^2} = 1 - 3\,\frac{\lambda_x\lambda_y + \lambda_x\lambda_z + \lambda_y\lambda_z}{(\lambda_x + \lambda_y + \lambda_z)^2} \quad (14)$$

It reflects both the symmetry and dimensionality of a microgel. This parameter is also limited between the values of 0 and 1. It reaches 1 when all beads located in a straight line and drops to zero for highly symmetric conformations.
Moreover, we calculate the distance between the centers of mass of precursor cores within the Janus-like microgel (Figure 3C V),

$$\Delta_{12} = \|\vec{r}_{1\,cm} - \vec{r}_{2\,cm}\| = \sqrt{\sum \left(r_{1\,cm_i} - r_{2\,cm_i}\right)^2},$$

$$i \in \{\mathrm{x, y, z}\} \quad (15)$$

Also, we compute the fraction of beads of microgels of each type (in regard to the total number of beads in the microgel) in its principal axis system into a slice thickness of 1σ as a function of z coordinate.

$$\rho_i(z) = \frac{N_i(z)}{N}, \qquad i \in \{\mathrm{O}, \mathrm{X}, \mathrm{I}^+, \mathrm{I}^-\} \tag{16}$$

After equilibration run, we collect statistics during 5·10^6 time steps for each system at each pH value. Every 1000 time steps, we measure all quantities introduced above characterized the geometry of the Janus-like microgel in its principal axis system. These ensemble averages will be denoted by overbar above a character; i.e., $\bar{a}$ is the time average of a

Results and Discussion

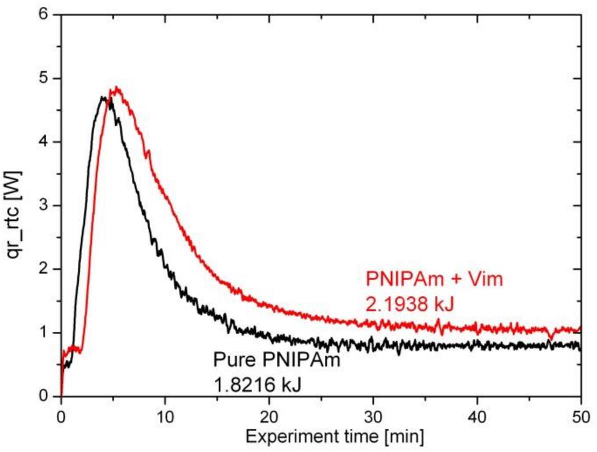

Figure S 1: Calorimetric measurements for pure NIPAm microgels (black) and NIPAm-VIm microgels (red). The numbers give the total reaction heat.

Figure S 1: Calorimetric measurements for pure NIPAm microgels (black) and NIPAm-VIm microgels (red). The numbers give the total reaction heat. Both curves have a similar shape, i.e., a fast increase in reaction heat within ~ 8 min, followed by a slower decay. Though the reaction is slightly longer for the copolymer than the homopolymer, both reactions are complete after ~ 30 min. Unexpectedly, it was not possible to copolymerize NIPAm with IA in the calorimeter (though the reaction worked well in a double-walled glass reactor). Instead of stable microgel dispersion, a sticky, hydrogel-like material was obtained. The reason for this is still unknown, though a reaction between IA and the metal probes is likely.

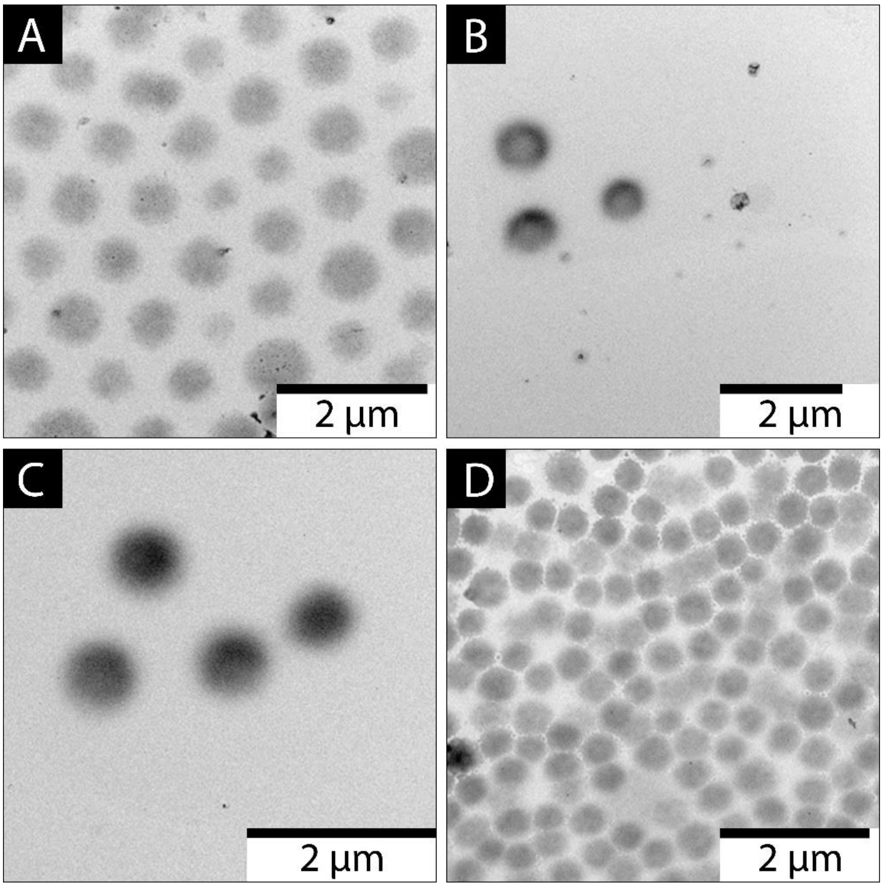

Figure S 2: TEM images of NIPAm-IA-VIm microgels obtained after t_p = 1 h and t_{mix} = (a) 1 min; (b) and (c) 3 min; (d) 5 min.

Figure S 2 presents the TEM images for a batch of samples prepared at t_p = 1 h. The images show that different t_{mix} lead to the formation of microgels with different distributions of IA. Mixing after 1 min leads to a homogenous distribution of IA in the microgels (Figure S2A). Mixing after 3 min results in microgels where one side is darker than the other (Figure S2B+C). t_{mix} > 3 min caused the formation of two separate types of microgels coexisting in the same sample. Since one type of microgel is slightly darker than the other, it can be assumed that separate NIPAm-VIm and NIPAm-IA microgels were formed (Figure S2D).

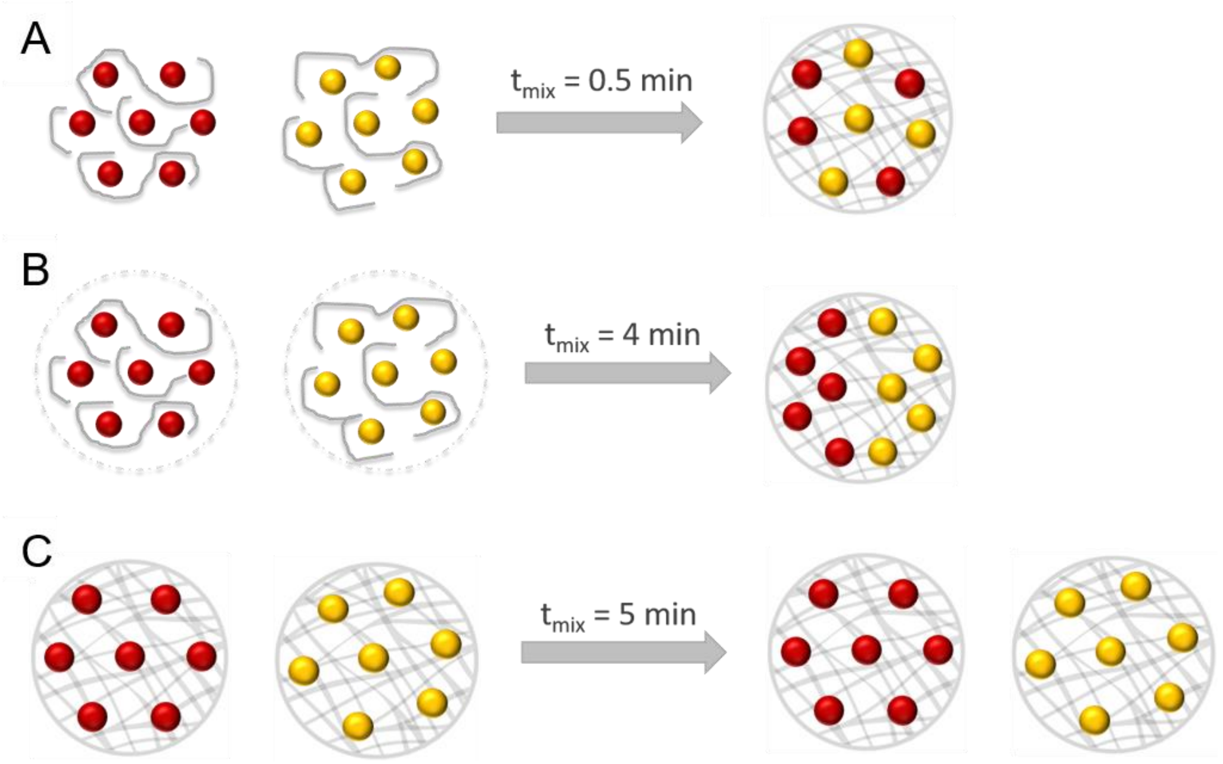

Figure S 3: The formation procedure of opposite charged precursor particles/microgels to different type of charged microgels. A) formation of mixed monomers to polyampholyte microgels with random dirstribution of ionizable groups. B) formation of pre-microgels into stable polyampholyte Janus-like polyampholyte microgels. C) formation of differently charged polyelectrolyte microgels.

As presented in Figure S 3, the precursor particles NIPAm-VIm and NIPAm-IA were initially prepared at different pH values to ensure the presence of charges during the mixing procedure (NIPAm-VIm at pH = 3, and NIPAm-IA at pH = 10). With different mixing time, different types of charged microgel systems can be created. With a mixing time of t_{mix} = 0.5 min (0.5 min for NIPAm-VIm and 2.5 min for NIPAm-IA), there is no clear formation of microgels yet (particle sizes around 30 nm for NIPAm-Vim and NIPAm-IA, see Figure 1). At this stage, once the reaction is mixed together containing all the unreacted monomers, the precursor particles prefer to gather together as in a normal precipitation polymerization process for polyampholyte microgels with random distribution of ionizable groups. For t_{mix} = 4 (4 min for NIPAm-VIm and 6.5 min for NIPAm-IA), the precursor particles already start to format into microgels, by mixing these pre-microgels with opposite charges, we strongly believe that according to the electrostatic driven forces, negative and positive charged pre microgels will come together, merge, and by further polymerization we achieve stable microgels with Janus-like structures. For t_{mix} = 5 (5 min for NIPAm-VIm and 7.5 min for NIPAm-IA), the precursor particles already formed stable microgels before mixing.

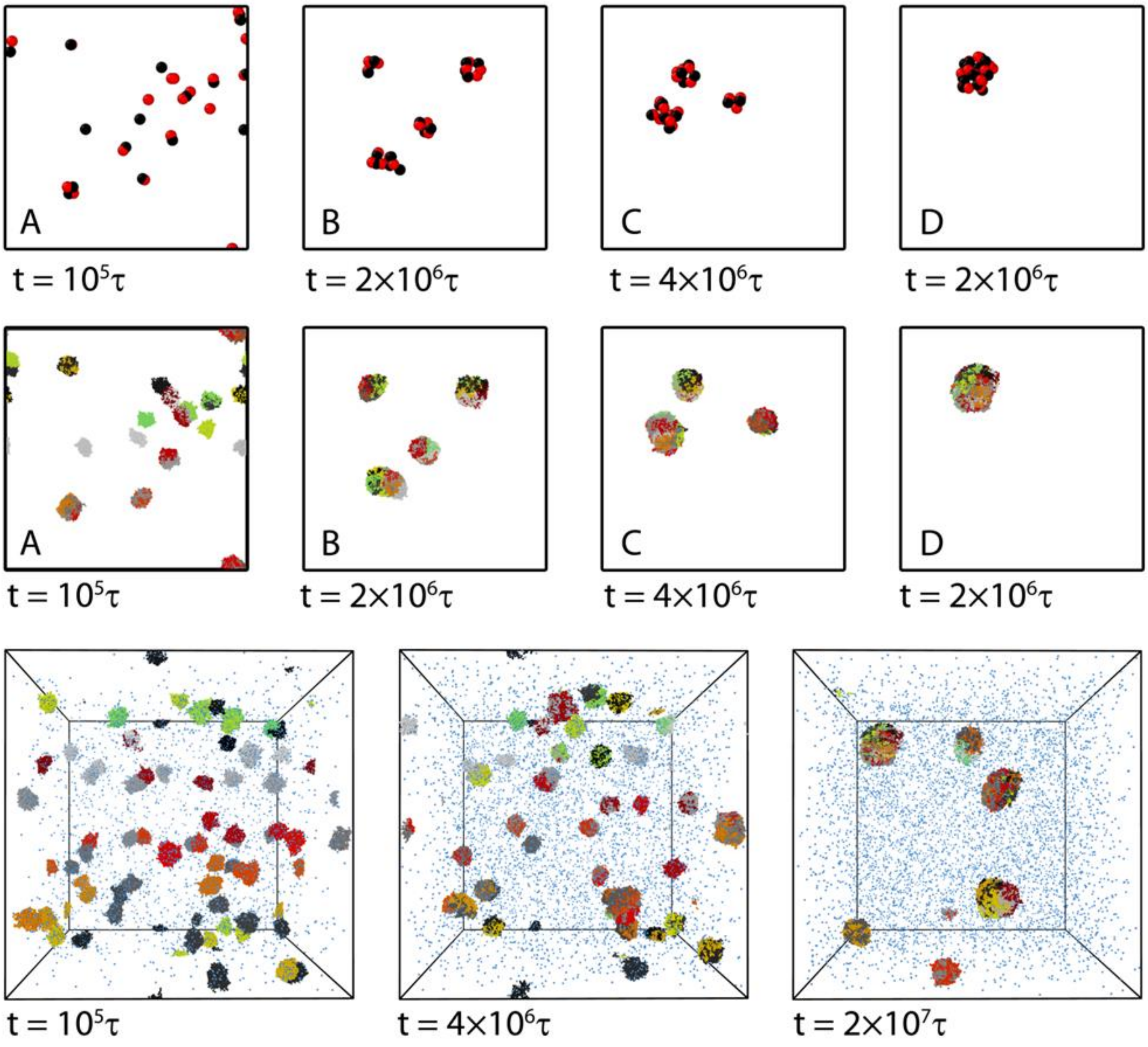

Figure S 4: Computer simulation: formation of microgels with a random distribution of ionizable groups from the mixture of polycationic and polyanionic pre-microgel nuclei. Case of short mixing time (System 1). The middle row shows only a few pre-microgel nuclei of the systems that, over time, form into large clusters. The top row shows the center of masses of the microgels plotted in the middle row. Colored microgel nuclei are polyanionic, while grayish microgel nuclei are polycationic. The number of cationic and anionic microgel nuclei before mixing are equal ($NC_{0.5k}$ = $NA_{0.5k}$ = 32). Blue dots correspond to the counterions.

In Figure S 4, the snapshots of the system at different timing during the equilibration are shown. All the polycationic microgel nuclei are plotted in grey, while all polyanionic microgel nuclei are presented in different bright colors. We chose such a color scheme to track the position and size of the individual microgels during their aggregation. We monitor the aggregation process by estimation the number of clusters of microgel nuclei in the simulation box, as shown in Figure S 5. Initially, at t = 0, all microgel nuclei were randomly distributed throughout the simulation box. We have 16, 32, and 64 clustersdepending on the system, where each cluster consists of single microgels nucleus. We observed, over a short time period after starting the simulation, that oppositely charged nuclei are attracted to each other to form "Janus like pairs" (dipoles, Figure S 4A top raw). The number of clusters is reduced by about half. The fastest formation of dipoles is observed for system 1, due to the higher diffusion rate of the small nuclei. Then the clusters growth occurs preferably by the electrostatic attraction between the dipole pairs, Figure S 4B top raw. Larger clusters are also formable by showing a quartet, sextet or octet structure. By increasing the time, these clusters stick together, to form a clusters of large orders Figure S 4C-D. Within the framework of the model, we cannot reliably establish the time at which one large cluster appears, because the diffusion rate of the clusters dramatically decreases with its growth. Nevertheless, it can be clearly observed that the formation of the statistically distributed microgels are caused by the forces of the electrostatic nature.

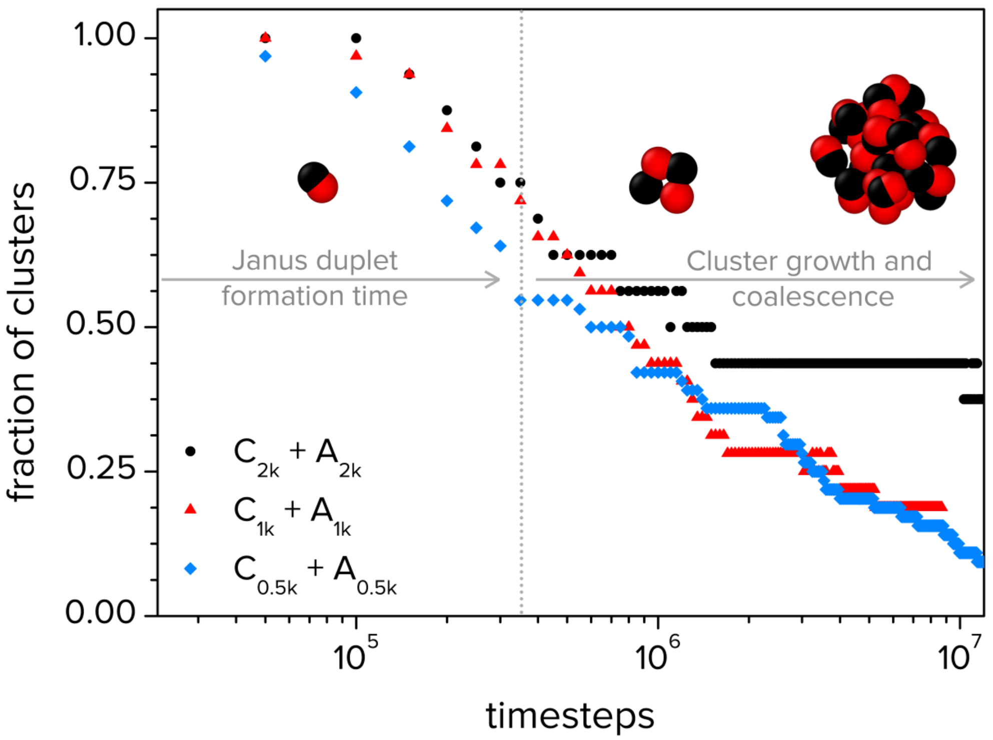

Figure S 5: Computer simulation: the fraction of clusters formed by the electrostatic interaction of oppositely charged pre-microgel nuclei as a function of time. Value 1.0 corresponds to the initial state of the system where all clusters consist of 1 microgel nucleus. The value 0.5 means that the number of clusters has halved due to its sticking. The number of cationic and anionic microgel nuclei before mixing are equal NC_{2k} = NA_{2k} = 8 (black circles), NC_{1k} = NA_{1k} = 16 (red triangles) and NC_{2k} = NA_{2k} = 8 (blue diamonds).

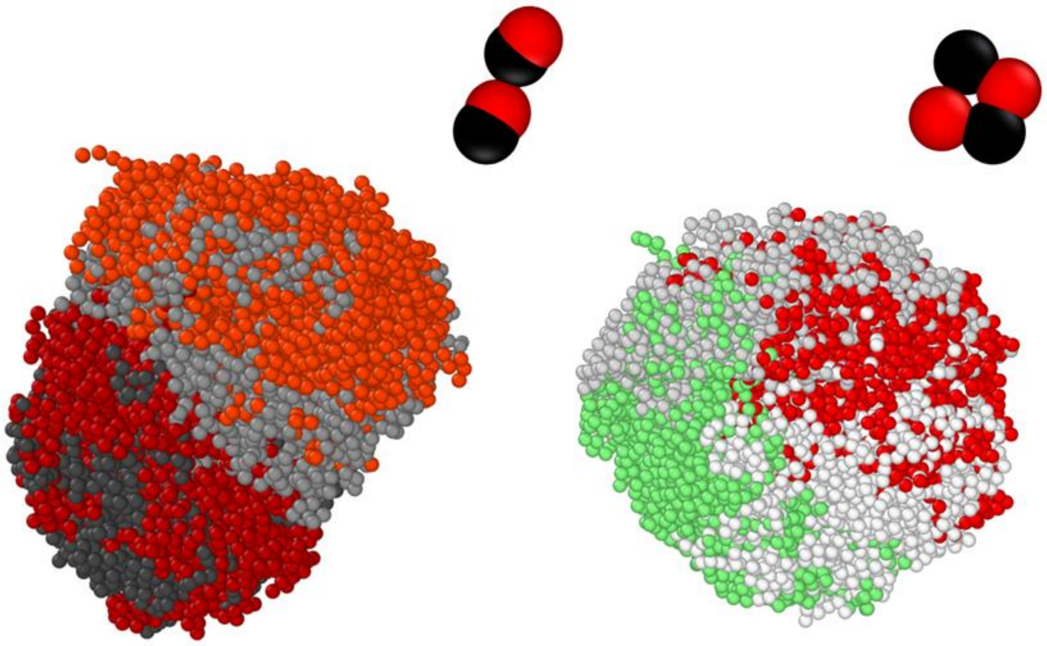

Figure S 6: Computer simulation: example of a quartet - the cluster formed by 4 oppositely charged pre-synthesized microgel nuclei. The number of cationic and anionic microgel inside the cluster is equal 2. Colored microgel nuclei are polyanionic, while grayish microgel nuclei are polycationic.

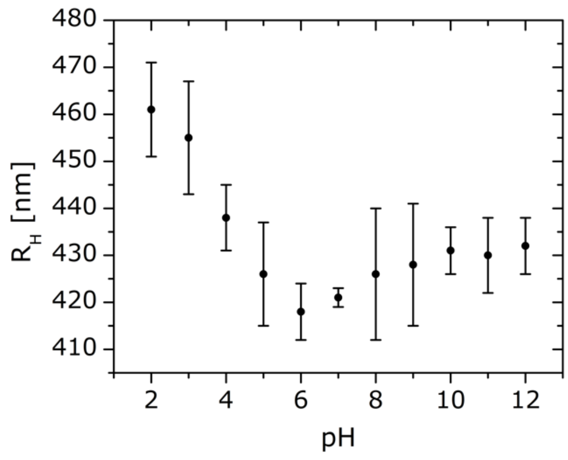

Figure S 7: Hydrodynamic radius R_H as a function of pH of Janus-like microgels at 20 °C.

Figure S 7: Hydrodynamic radius RH as a function of pH of Janus-like microgels at 20 °C. presents the pH sensitivity of the Janus-like microgels measured with Zetasizer NanoZS (Malvern, UK). The size of the microgels was measured at different pH from 2 to 12, using self-prepared buffer solutions with an ionic strength of 30 mM at 20 °C. However, the accuracy of the size measurements here should be treated with caution, since it is known that the salt concentration will screen the charges of weak polyelectrolytes and thus affect the particle sizes. Additionally, the particle sizes were measured at an angle of 173° in the Zetasizer, compared to 90° in the ALV. Nevertheless, Figure S 7: Hydrodynamic radius RH as a function of pH of Janus-like microgels at 20 °C. should give a general trend of the microgels sizes at different pH values.

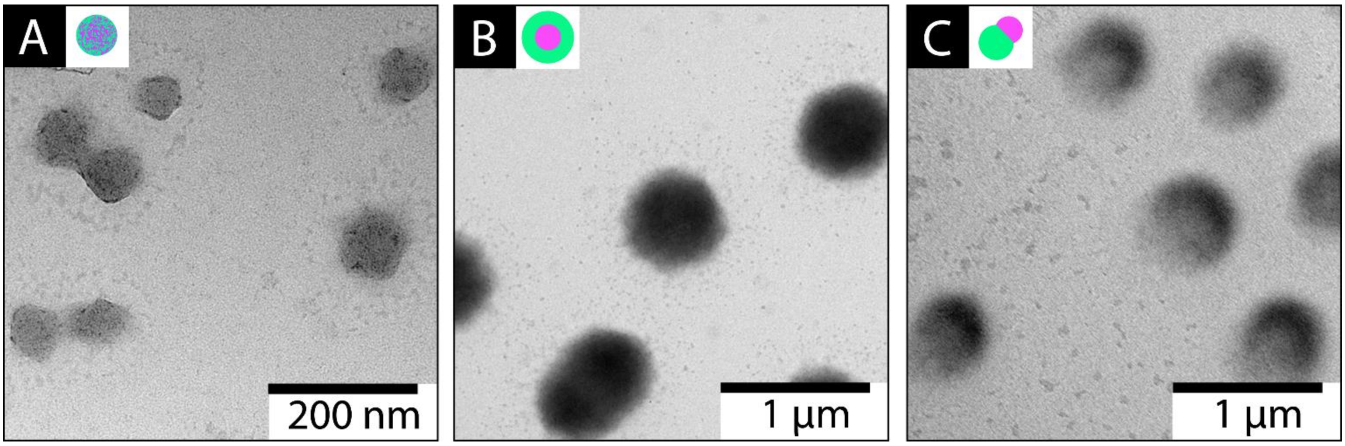

Figure S 8: TEM images of the morphology of polyampholyte microgels with (A) random, (B) core-shell, and (C) Janus-like distribution.

Figure S 8 presents the morphology of differently structured polyampholyte microgels was detected using TEM. All samples were stained with $U(Ac)_3$, and the measurements were carried out at 20 °C, and pH = 6

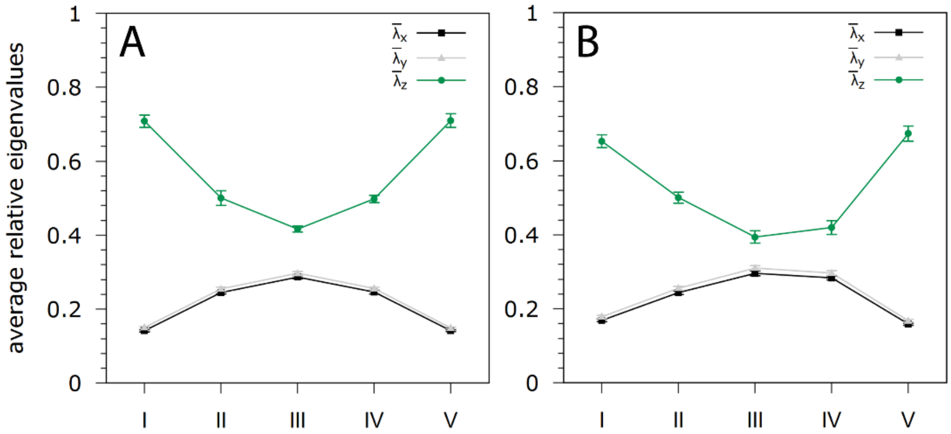

Figure S 9: Three eigenvalues of the average instantaneous radius of gyration tensor, divided by the mean-square radii of gyration, vs. the different ratio of ionized groups IA:VIm within the microgels: I – 0%:10%; II – 2.5%:7.5%; III – 5%:5%; IV – 7.5%:2.5%; V – 10%:0%. Vertical bars denote standard deviations of the mean values. Cases of (A) Symmetric and (B) Asymmetric Janus-like microgels.

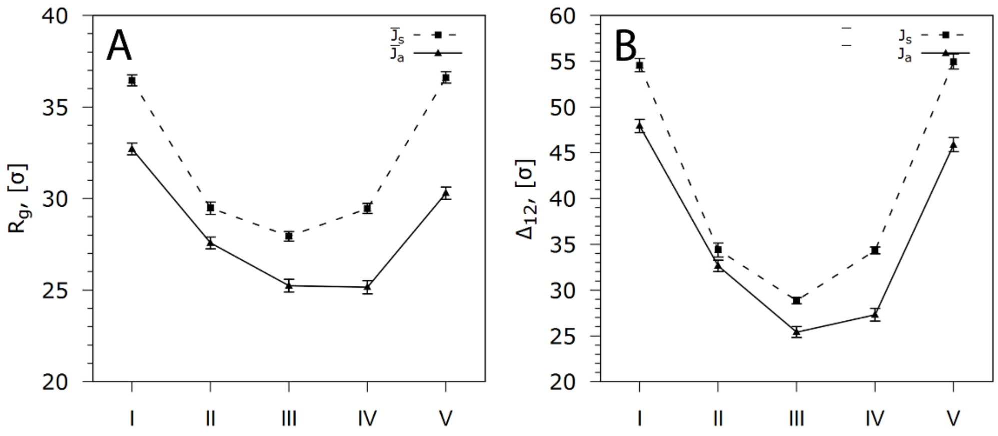

Figure S 10: (A) Average radius of gyration and (B) distance between the centers of mass of precursor cores within the Janus-like microgel as the functions of the different ratio of ionized groups IA:Vim within the microgels: I – 0%:10%; II – 2.5%:7.5%; III – 5%:5%; IV – 7.5%:2.5%; V – 10%:0%. Vertical bars denote standard deviations of the mean values.

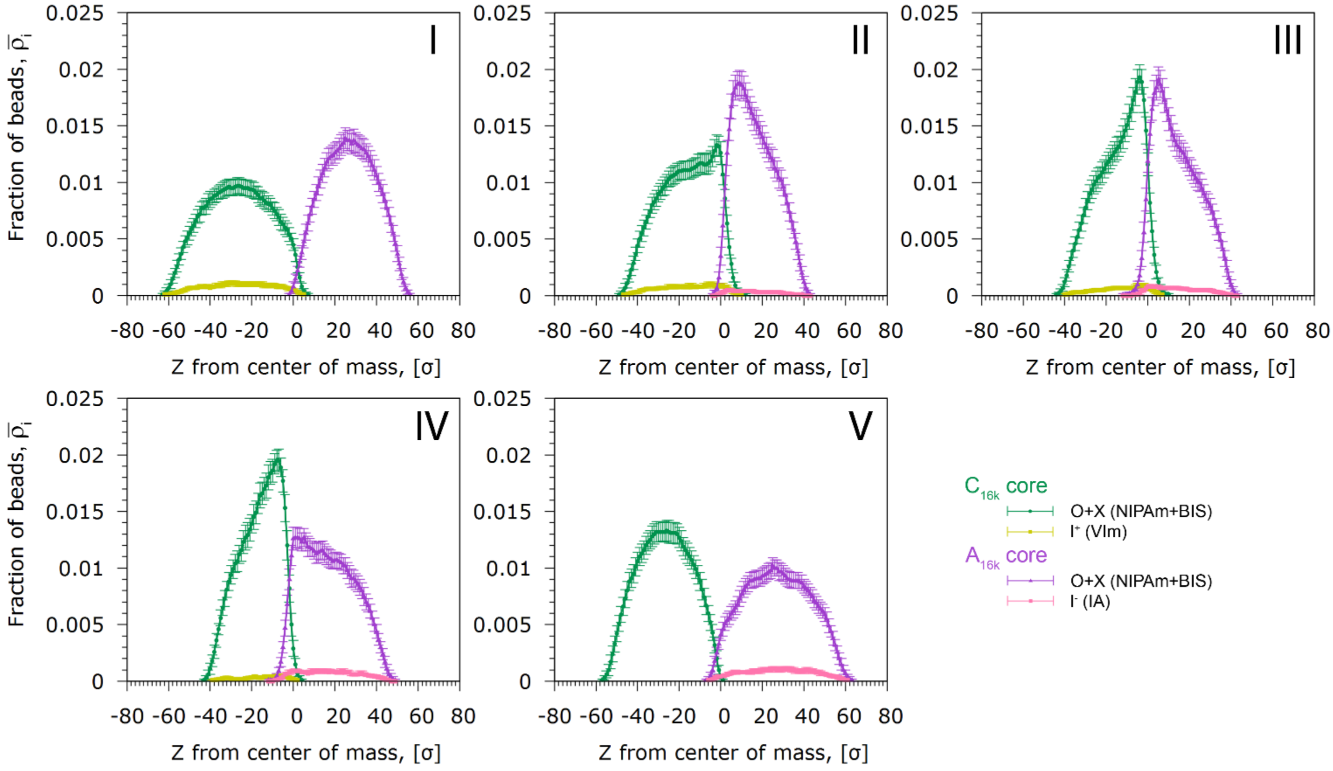

Figure S 11: The fraction of beads of symmetric Janus-like microgels, Js (with respect to the total number of beads in the microgel) in a slice thickness of 1σ as a function of Z coordinate in its principal axis system. I, II, III, IV, and V denote the different ratio of ionized groups IA:VIm within the microgels: I – 0%:10%; II – 2.5%:7.5%; III – 5%:5%; IV – 7.5%:2.5%; V – 10%:0%. Vertical bars denote standard deviations of the mean values.

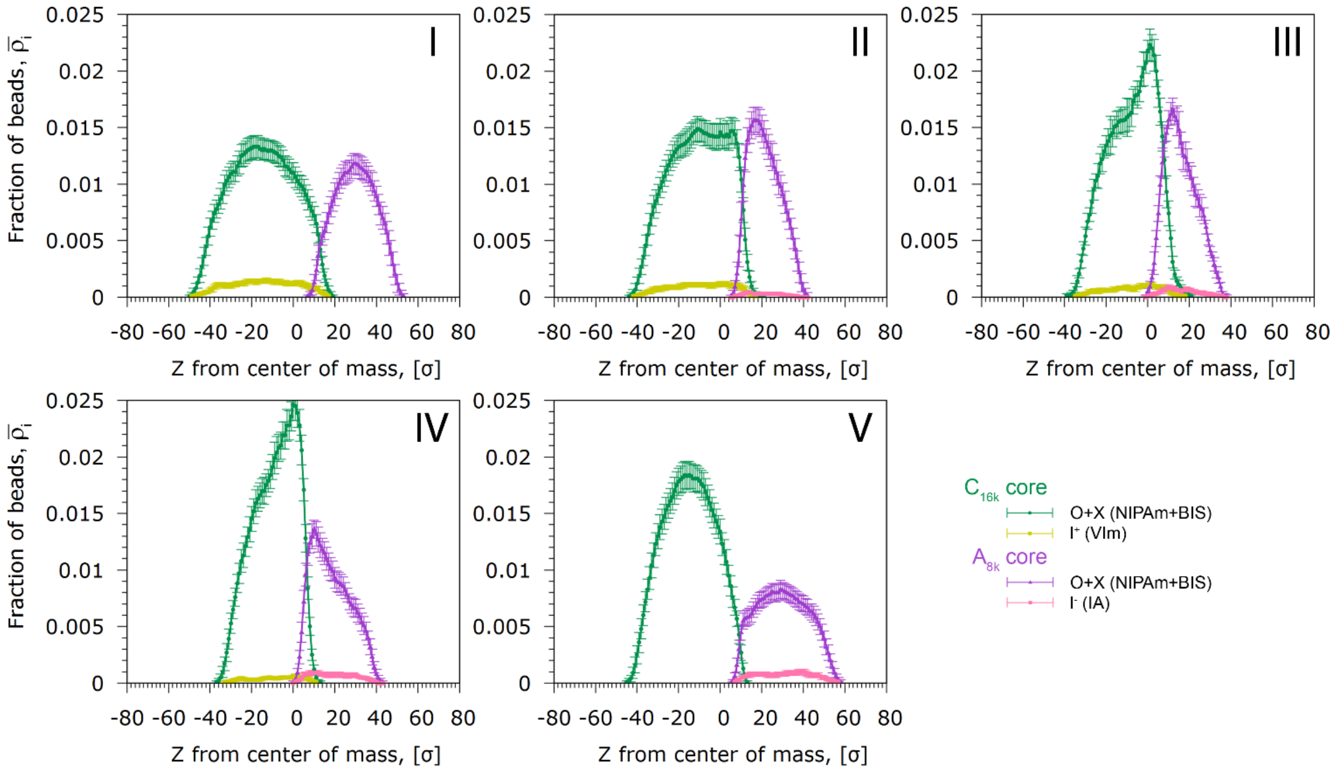

Figure S 12: The fraction of beads of asymmetric Janus-like microgels, Ja (with respect to the total number of beads in the microgel) in a slice thickness of 1σ as a function of Z coordinate in its principal axis system. I, II, III, IV, and V denote the different ratio of ionized groups IA:VIm within the microgels: I – 0%:10%; II – 2.5%:7.5%; III – 5%:5%; IV – 7.5%:2.5%; V – 10%:0%. Vertical bars denote standard deviations of the mean values.

Figure S 13 and S 14 present the particle form factors of the microgels at pH = 2, 6 and 9. The data was obtained via static light scattering and are evaluated by fitting the data using the fuzzy sphere model.[7] This model describes the radial scattering length density by a box function convoluted with a Gaussian to account for the surface fuzziness of the microgels, which is obtained due to the faster reaction kinetics of the cross-linking agent BIS compared to the main monomer NIPAm.[15] As a result, the form factor can be described as follows:

$$P_{inhom}(q) = \left[\frac{3(\sin(qR) - qR\cos(qR))}{(qR)^3} \cdot \exp\left(-\frac{(\sigma_{surf}q)^2}{2}\right)\right]^2 \quad (17)$$

R describes the radius of the microgel, q is the scattering vector and σ_{surf} accounts for the width of the surface fuzziness.

For the case of spherical microgels, we expect the model to fit the data appropriately. In Figure S13, the scattering intensity is plotted against the scattering vector. For pH = 6 the fit based on the fuzzy sphere model matches the data and the physical parameters (R_{SLS} = 334 ± 7 nm for λ = 640 nm and R_{SLS} = 290 ± 20 nm λ = 407 nm, compared to R_H = 419 ± 11 nm). For pH = 2 and pH = 9 two fits are shown: First, all parameters were released to find a fit based on the fuzzy sphere model best matching the data (in Figure S13 = “data matching”). For pH = 9, this results in a radius of 325 ± 11 nm compared to a hydrodynamic radius of 479 ± 7 nm (R_{SLS} = 319 ± 1 nm at λ = 407 nm). Compared to the ratio of R_{SLS}/ R_H = 0.80 for pH = 6, a ratio of 0.68 for pH = 9 is irrational. In a second attempt, the radius for fitting was fixed to a more reasonable value of 374 nm (≈ 0.78 x 479 nm, in Figure S13 = “parameters matching”). For pH = 2, a radius of 508 ± 1 nm (for both laser wavelengths) was obtained for the data matching fit, compared to an R_H = 433 ± 11 nm. Given a larger radius obtained via SLS compared to DLS, the fit is physically unreasonable here. For the parameters matching fit, the radius was fixed to 346 nm (≈ 0.82 x 433 nm). The fact that the data matching fits of the particle form factors do not result in physically reasonable results for pH = 2 and pH = 9, and the clear mismatching of the parameters matching fits indicates a non-spherical shape of the microgels. This finding backs the observation from microscopy images and computer simulations that the microgels are non-spherical for pH values, where either one side is charged.

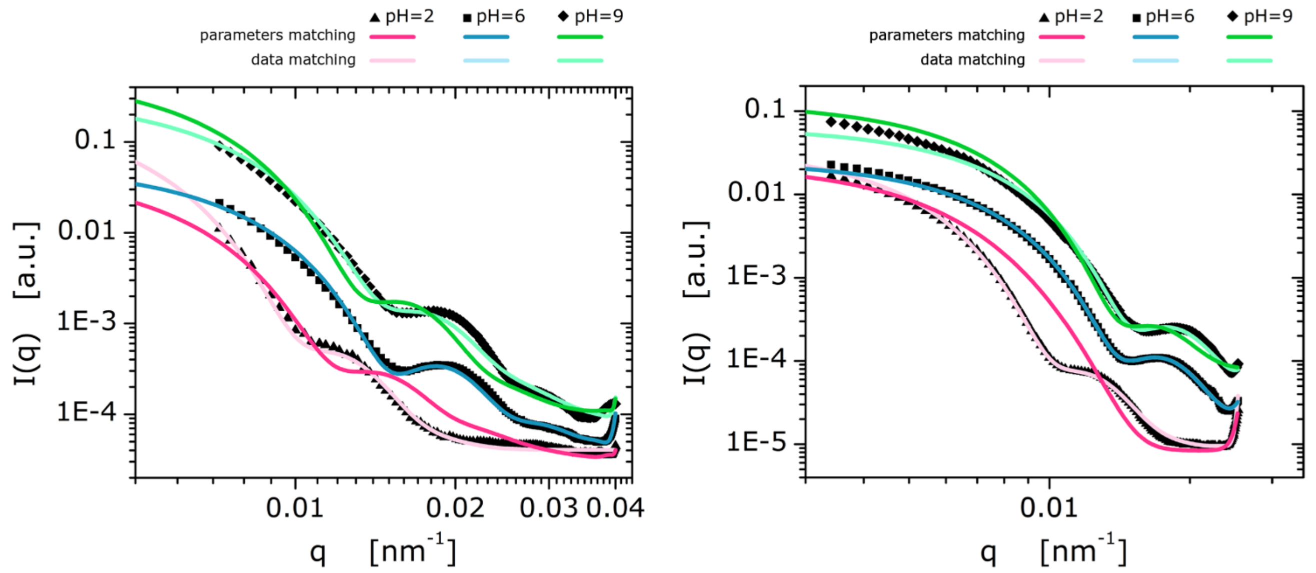

Figure S 13: Particle form factors obtained via SLS using a laser wavelength of λ = 407 nm (left) and λ = 640 nm (right). Experimental data are shown for pH = 2, pH = 6 and pH = 9 at T = 20 °C. The corresponding fits are based on the fuzzy sphere model and are either matching the physical parameters or the experimental data (pH = 2 and 9), or both (pH = 6).

For pH = 6, the microgel is expected to be homogenously swollen since at this point charge neutrality is achieved. Due to this, the microgel should be thermo-responsive at this pH–value. Figure S 14 shows particle form factors of the microgel at pH = 6 in the swollen and the collapsed state obtained via SLS using different laser wavelengths. The data was fitted based on the fuzzy sphere model. At a laser wavelength of λ = 407 nm, the radius decreases from R_{SLS} = 290 ± 20 nm to R_{SLS} = 180 ± 2 nm. The deviation of the radius obtained for the swollen state between the two laser wavelength is conspicuous. However, the error of 20 nm for the fit at λ = 407 is relatively high, but multiple measuring resulted in similar results. For λ = 640 nm, similar results are obtained. The radius changes from R_{SLS} = 334 ± 7 nm at T = 20 °C to R_{SLS} = 186 ± 4 nm at T = 50 °C.

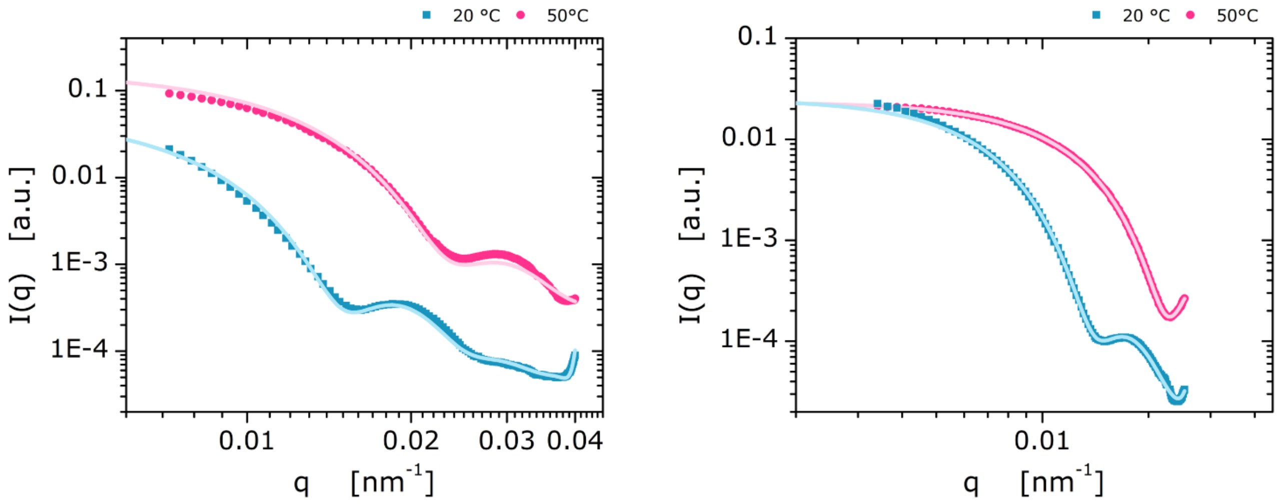

Figure S 14: Particle form factors obtained via SLS using a laser wavelength of λ = 407 nm (left) and λ = 640 nm (right). Experimental data are shown for pH = 6 in the swollen state at T = 20 °C and the collapsed state at T = 50 °C. The corresponding fits are based on the fuzzy sphere model

In order to get a first idea on the size of the microgels, DLS measurements were conducted for samples at pH = 2, 6 and 9 at T = 20 °C and additionally at T = 50 °C for pH = 6. The corresponding fits of the decay rate of a second-order cumulant fit against q^2 are shown in Figure S 15.[8] The slope of the linear fits corresponds to the diffusion coefficients. Based on the Stokes-Einstein equation hydrodynamic radii of R_H (pH = 2, 20 °C) = 433 ± 11 nm, R_H (pH = 9, 20 °C) = 479 ± 7 nm, R_H (pH = 6, 20 °C) = 419 ± 11 nm and R_H (pH = 6, 50 °C) = 225 ± 1 nm were calculated.[9]

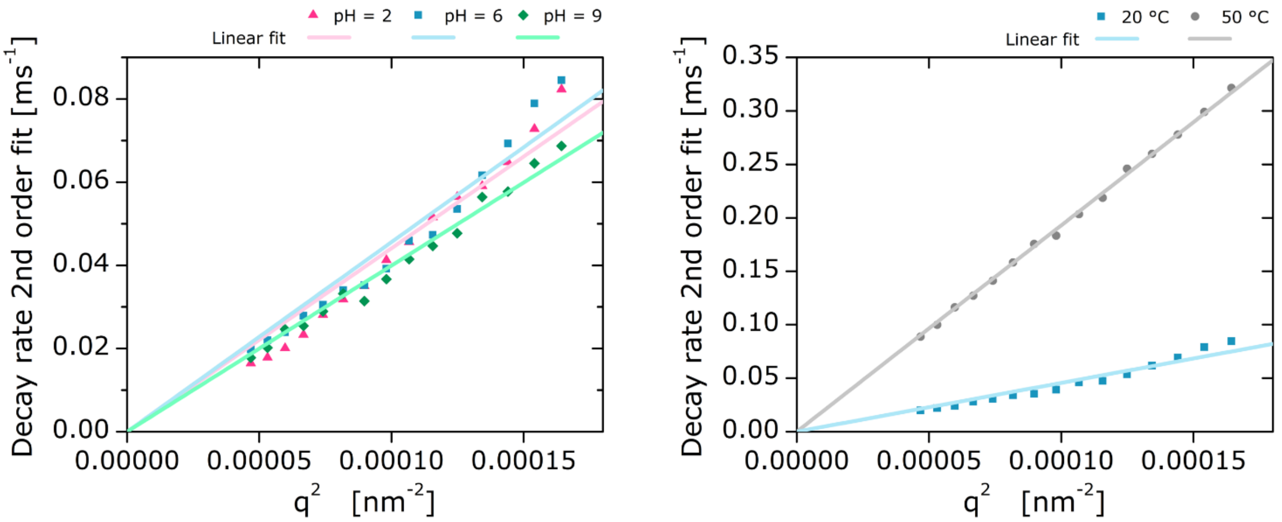

Figure S 15*:* Plot of the decay rate of a second order cumulant fit against q^2 for the microgels at pH = 2, 6 and 9 and T = 20 °C (left) and pH = 6 at T = 20 °C and T = 50 °C (right).

References

[1] R. Schroeder, A. A. Rudov, L. A. Lyon, W. Richtering, A. Pich, I. I. Potemkin, *Macromolecules* **2015**, *48*, 5914.
[2] R. Pelton, *Adv. Colloid Interface Sci.* **2000**, *85*, 1.
[3] S. Plimpton, *J. Comput. Phys.* **1995**, *117*, 1.
[4] a) G. C. Claudio, K. Kremer, C. Holm, *J. Chem. Phys.* **2009**, *131*; b) A. M. Rumyantsev, A. A. Rudov, I. I. Potemkin, *The Journal of Chemical Physics* **2015**, *142*, 171105.
[5] K. Kremer, G. S. Grest, *The Journal of Chemical Physics* **1990**, *92*, 5057.
[6] S. Toxvaerd, J. C. Dyre, *The Journal of Chemical Physics* **2011**, *134*, 081102.
[7] O. L. J. Virtanen, **2015**.
[8] W. Burchard, W. Richtering*, Vol. 80*, Steinkopff, Darmstadt, **1989**, pp. 151.
[9] A. Einstein, *Annalen der Physik* **1905**, *322*, 549.
[10] M. Ovesny, P. Krizek, J. Borkovec, Z. Svindrych, G. M. Hagen, *Bioinformatics* **2014**, *30*, 2389.
[11] M. El Beheiry, M. Dahan, *Nat. Methods* **2013**, *10*, 689.
[12] E. Gau, D. M. Mate, Z. Zou, A. Oppermann, A. Töpel, F. Jakob, D. Wöll, U. Schwaneberg, A. Pich, *Biomacromolecules* **2017**, *18*, 2789.
[13] D. N. Theodorou, U. W. Suter, *Macromolecules* **1985**, *18*, 1206.
[14] R. P. Smith, E. M. Mortensen, *The Journal of Chemical Physics* **1960**, *32*, 502.
[15] M. Stieger, W. Richtering, J. S. Pedersen, P. Lindner, *J. Chem. Phys.* **2004**, *120*, 6197.

Author Contributions

W. Xu performed the experiments, analyzed the *in-situ* DLS, Calorimeter, temperature- and pH-responsive DLS curves, the TEM images, and wrote the manuscript. Dr. A. Rudov performed the simulations, graphic design and wrote the simulation part of the manuscript. A. Oppermann performed the SFM images. S. Wypysek performed the SLS and DLS experiments, analyzed the curves, and wrote the SLS/DLS part in the manuscript. M. Kather helped to perform the Calorimeter measurements and proofread the manuscript. Dr. R. Schroeder performed preliminary test experiments. Prof. A. Pich, Prof. W. Richtering, Prof. I. I. Potemkin, and Prof. D. Wöll conceived the concept, discussed the results and wrote the manuscript. All authors provided input and feedback for manuscript preparation. All authors approved the final version of the manuscript.
